# Continuous monitoring of neutrophils to lymphocytes ratio for estimating the onset, severity, and subsequent prognosis of immune related adverse events

**DOI:** 10.1038/s41598-020-79397-6

**Published:** 2021-01-14

**Authors:** Ryosuke Matsukane, Hiroyuki Watanabe, Haruna Minami, Kojiro Hata, Kimitaka Suetsugu, Toshikazu Tsuji, Satohiro Masuda, Isamu Okamoto, Takashi Nakagawa, Takamichi Ito, Masatoshi Eto, Masaki Mori, Yoichi Nakanishi, Nobuaki Egashira

**Affiliations:** 1 Department of Pharmacy, Kyushu University Hospital, 3-1-1 Maidashi, Higashi-ku, Fukuoka, 812-8582 Japan; 2 Department of Clinical Pharmacology and Biopharmaceutics, Graduate School of Pharmaceutical Sciences, Kyushu University, Fukuoka, Japan; 3 Department of Clinical Pharmacy, Faculty of Pharmaceutical Sciences, International University of Health and Welfare Narita Hospital, Chiba, Japan; 4 Research Institute for Diseases of the Chest, Graduate School of Medical Sciences, Kyushu University, Fukuoka, Japan; 5 Department of Otorhinolaryngology, Graduate School of Medical Sciences, Kyushu University, Fukuoka, Japan; 6 Department of Dermatology, Graduate School of Medical Sciences, Kyushu University, Fukuoka, Japan; 7 Department of Urology, Graduate School of Medical Sciences, Kyushu University, Fukuoka, Japan; 8 Department of Surgery and Science, Graduate School of Medical Sciences, Kyushu University, Fukuoka, Japan

**Keywords:** Biomarkers, Oncology

## Abstract

Immune checkpoint inhibitors (ICIs) play a central role in various cancers. ICIs can cause immune-related adverse events (irAEs). As severe irAEs can be life-threatening, biomarkers for estimating irAE onset are crucial. The neutrophils-to-lymphocytes ratio (NLR) reflects the systemic immune condition and known as a prognostic marker in ICI treatment. Our study evaluated if the NLR corresponded with irAEs, and its feasibility as a biomarker for irAE onset. We retrospectively analyzed 275 cancer patients treated with anti-PD-1 monotherapy. We observed 166 irAEs in 121 patients. The NLR was significantly elevated during irAEs. Patients experiencing interstitial pneumonitis showed NLR elevation 4 weeks before initial symptoms and diagnosis. Analyzing receiver operating characteristics curves revealed that elevated NLR distinguished subsequent pneumonitis severity with high accuracy (AUC 0.93, sensitivity 88.9%, specificity 88.2%, cut-off 2.37, *p* = 0.0004). After a severe irAE occurred, two NLR trends were observed. Patients who showed a prompt reduction in elevated NLRs had favorable progression-free survival (hazard ratio 0.32, 95% CI 0.10–1.01, p = 0.0140) and overall survival (hazard ratio 0.23, 95% CI 0.06–0.86, *p* = 0.0057) compared to the patients who maintained elevated NLRs. These findings suggest that continuous monitoring of NLR trends may predict irAE onset and severity and subsequent prognosis.

## Introduction

Programmed cell death-1 (PD-1), its associated ligand (PD-L1), and cytotoxic T-lymphocyte associated antigen-4 (CTLA-4) negatively regulate immune function and are constitute targets for cancer immunotherapy^1^. Blocking these molecules via immune checkpoint inhibitors (ICIs) has been shown to activate autoimmunity against tumors, thereby exerting an anti-tumor effect^2^. The PD-1 inhibitors nivolumab and pembrolizumab have been approved for treating various cancer types and have significantly improved therapeutic outcomes^3–7^. However, treatment with ICIs has been reported to lead to the development of severe adverse events associated with autoimmunity, which do not occur with conventional chemotherapy. These effects are known as immune-related adverse events (irAEs), and such activated immune function has been shown to damage healthy organs throughout the body^8,9^. The most common irAEs are skin disorders, endocrine dysfunction, hepatitis, colitis, and interstitial pneumonitis^10^. As the development of severe irAEs may become life-threatening, numerous screening tests are recommended to enable early detection and treatment interventions^11–13^.

The development of irAEs is a favorable prognostic factor when treating various cancers with ICIs^14–16^; therefore, irAEs and tumor suppression may share common mechanisms of the activated immune system^17^. Studies have been conducted to identify the biomarkers that detect an activated immune response since the development of treatments with ICIs^18^. For example, the positive expression of PD-L1 in tumors, frequency of myeloid-derived suppressor cells, and regulatory T-cells in the blood are associated with increased overall survival (OS) in melanoma^19–21^. Apart from the therapeutic effects of ICIs, several reports have described molecular profiles associated with irAEs. Specific *HLA* genes have been reported as a risk factor for the development of irAEs^22,23^. Chemokines, such as soluble levels of cluster of differentiation 163 (CD163) and C-X-C motif chemokine ligand 5 (CXCL5), elevate after treatment with ICIs and relate to irAEs^24^. These biomarkers may be useful for predicting irAEs. However, their determination often requires additional blood samples and special equipment. Further validation studies are required before these potential biomarkers can be applied in clinical practice.

Activation of immune cells and physiological conditions are reflected in laboratory data, which provide routinely medical information. These data are derived from already validated clinical tests such as total blood cell counts, serum chemistry, coagulation factors, hormones, tumor markers, and physical measurements^25^. For example, a previous report showed that blood cell counts such as lymphocytes and eosinophils were associated with the survival of patients treated with ipilimumab and pembrolizumab^21,26^. Moreover, the neutrophil-to-lymphocyte ratio (NLR) is a useful indicator of the systemic immune condition during cancer treatment, and several studies have suggested that the pre-treatment NLR is a significant prognostic factor during treatment with ICIs^27–29^. However, few studies have demonstrated the usefulness of these factors in the investigation of the development of irAEs^30–32^. If these data can effectively predict irAE onset, severity, and target organs, they will have immense clinical significance.

ICIs are widely used in various tumor treatments. Concomitant treatment with ICI drugs that target other immune checkpoint molecules, radiotherapy, as well as conventional cytotoxic and molecular targeting drugs have been studied^33^; moreover, drug discovery for other immune checkpoint molecules is ongoing^34^. Studies of irAEs are important for predicting the safety of ICIs. In this study, we retrospectively evaluated real-world data acquired from a registry of ICI-treated patients, including patient background and routinely acquired laboratory data. We estimated the development of irAEs using clinically accessible biomarkers, mainly focusing on the levels of neutrophils, lymphocytes, and the NLR.

## Methods

### Ethics declarations

This study was conducted in accordance with the Declaration of Helsinki. Ethical approval was provided by the Institutional Review Boards of the Kyushu University Graduate School and Faculty of Medicine (Approval No. 2020-155). Owing to the retrospective nature of this study, informed consent was waived, and our official website was used as an opt-out method, which was also approved by the Institutional Review Boards of the Kyushu University Graduate School and Faculty of Medicine.

### Study design

We retrospectively analyzed patients with metastatic or unresectable solid tumors (namely, non-small cell lung cancer (NSCLC), malignant melanoma, head and neck cancer (HNC), and renal cell carcinoma (RCC)) at Kyushu University Hospital in Japan. In this study, all of the patients were Asian. Patients treated with at least one infusion of nivolumab or pembrolizumab monotherapy from September 2014 until December 2018 were eligible. The cut-off date for data collection was May 31, 2020. Regardless of the clinical practice or trial, patients who had received the anti-CTLA4 antibody, anti-PD-L1 antibody, or any combination therapy that included ICIs were excluded from our analysis. Patients with Eastern Cooperative Oncology Group Performance Status (ECOG PS) 3 or 4 were also excluded from this study as recommendations suggest that these patients should not receive systemic treatments including ICIs.

### Data acquisition

We extracted de-identified clinical information including primary tumor, number of metastases, prior treatment, treatment duration, outcome, irAE incidences, physical information, social history, and clinical laboratory data from electronic medical records. Laboratory data were obtained every visit and before ICI administration, but the interval differed due to administered ICI drugs, treatment plans, and conditions specific to each patient. The NLR was calculated from the number of absolute neutrophil counts divided by the number of absolute lymphocyte counts. To objectively diagnose the irAEs, suitable examinations were conducted as needed. For example, bronchoscopies and alveolar lavage fluid collections were performed to confirm pneumonitis, provocative loading tests were performed to diagnose hypophysitis, total colonoscopies were performed to test for colitis, and biopsy samples of irAE-afflicted organs (e.g., skin, kidneys, and liver) were evaluated by a pathologist to rule out other possibilities Moreover, these diagnoses were double-checked by the authors during data collection. Grading of these irAEs was determined using the National Cancer Institute Common Terminology Criteria for Adverse Events v.5.0.

### Statistical analysis

Statistical comparisons were made using the Wilcoxon matched-pairs test, Mann–Whitney test, and paired *t*-tests for two-group comparisons. One-way ANOVA with Tukey’s post hoc test was performed for multiple group comparisons. One-way ANOVA using a mixed-effects model followed by Holm-Sidak’s post hoc test was used for repeated measures analysis such as the sequential evaluation of laboratory data. Survival probabilities were analyzed using the Kaplan–Meier method, and different groups were compared using the log-rank test. Overall and prognostic-free survival was calculated from the start of the ICI treatment until the date of events. Multivariate analysis was performed using multiple logistic regression analysis and the Cox proportional hazards model. In receiver operating characteristics (ROC) curve analysis, cut-off values were determined using the highest Youden index. A comparison of clinical variables in patients dichotomized by the NLR trend was examined with Fisher’s exact test. All quantification, calibration, and statistical analyses were carried out using Prism version 8.0.1 (GraphPad Software, La Jolla, CA, USA) and JMP version 14.2.0 (SAS Institute Inc., Cary, NC, USA).

## Results

### Background of patients

We collected data from 275 patients with recurrent or unresectable solid tumors who were treated with nivolumab or pembrolizumab (Table 1). Since we aimed to analyze irAEs induced via anti-PD-1 antibody monotherapy, patients who had been treated with either anti-CTLA-4 (ipilimumab) antibody, anti-PD-L1 (avelumab, atezolizumab, and durvalumab) antibody, or any combination therapy were excluded from the study. The median age of patients was 68 years (16–89 years), and 73.8% were male. Primary tumors included malignant melanoma (n = 55, 20.0%), NSCLC (n = 119, 43.3%), HNC (n = 63, 22.9%), and RCC (n = 38, 13.8%). In particular, 218 (79.3%) and 57 (20.7%) patients had been treated with nivolumab and pembrolizumab, respectively.

**Table 1:** Patient background.

	Total	Melanoma	NSCLC	HNC	RCC
	n = 275, (%)	n = 55, (%)	n = 119, (%)	n = 63, (%)	n = 38, (%)
**Age (years), median (range)**	68 (16–89)	70 (16–86)	68 (39–89)	66 (42–81)	69 (52–83)
**Sex**					
Male	203 (73.8)	30 (54.5)	98 (82.4)	46 (73.0)	29 (76.3)
Female	72 (26.2)	25 (45.5)	21 (17.6)	17 (27.0)	9 (23.7)
**ECOG PS**					
0–1	251 (91.3)	53 (96.4)	108 (90.8)	56 (88.9)	34 (89.5)
2	24 (8.7)	2 (3.6)	11 (9.2)	7 (11.1)	4 (10.5)
**Number of prior regimens**					
0	67 (24.4)	23 (41.8)	23 (19.3)	20 (31.7)	1 (2.6)
1	114 (41.4)	23 (41.8)	46 (38.7)	30 (47.6)	15 (39.5)
≥ 2	94 (34.2)	9 (16.4)	50 (42.0)	13 (20.6)	22 (57.9)
**Number of metastatic sites**					
0	24 (8.7)	2 (3.6)	4 (3.4)	18 (28.6)	0 (−)
1	103 (37.5)	20 (36.4)	43 (36.1)	30 (47.6)	10 (26.3)
≥ 2	148 (53.8)	33 (60.0)	72 (60.5)	15 (23.8)	28 (73.7)
**Anti-PD-1 antibody**					
Nivolumab	218 (79.3)	41 (74.5)	76 (63.9)	63 (100)	38 (100)
Pembrolizumab	57 (20.7)	14 (25.5)	43 (36.1)	0 (−)	0 (−)

All patients are Asian.

*NSCLC* non-small cell lung cancer, *HNC* head and neck cancer, *RCC* renal cell carcinoma, *ECOG PS* Eastern Cooperative Oncology Group performance status, *PD-1* programmed cell death-1.

### Incidences of irAEs

Throughout the observation period, 166 irAEs were reported in 121 patients (44.0%), with 29 patients (10.5%) experiencing irAEs of Grade 3 or higher. During the observation period, 86 patients experienced at least one irAE, 26 patients had two irAEs, and nine patients had more than three irAEs. The median duration of the first irAE incidence was 53 days (3–711 days) after the initiation of treatment. The irAEs that occurred early (within a week) after the start of ICIs included neuropathy, rash, liver dysfunction, and colitis. The detailed information of the developed irAEs are summarized (Table 2). Common irAEs were rashes (44 cases), pneumonitis (26 cases), thyroiditis (17 cases), pruritus (11 cases), hypophysitis (12 cases), and colitis (9 cases). Other incidences included hematopoietic abnormalities, such as anemia, neutropenia, thrombocytopenia (4 cases), type 1 diabetes mellitus, secretion of inappropriate antidiuretic hormone with hyponatremia (3 cases), uveitis, myocarditis, oral mucositis, asymptomatic amylase, and lipase elevation, dysgeusia, stomach discomfort (2 cases), and bullous pemphigoid (1 case). Corticosteroid treatment and hormone replacement therapy was conducted in 47 (25.9%) and 31 (18.7%) of the irAEs, respectively. For one case in pneumonitis, cyclophosphamide was used as an intensive immunosuppressant therapy. Comparing the incidences of irAEs by the type of primary tumor showed that 63 patients (52.9%) with NSCLC developed irAEs, among which 13 patients (10.9%) were Grade 3 or higher. In contrast, only 20 patients (31.7%) with HNC experienced irAEs, with Grade 3 or more severe events observed in seven patients (11.1%) (Supplementary Table S1). Thus, severe incidences of irAEs occurred nearly invariably across all cancer types examined.

**Table 2:** Immune-related adverse events of this study population.

	n	Median duration		Grade 1, n (%)	Grade 2, n (%)	≥ Grade 3, n (%)	Corticosteroid treatment*, n (%)	Hormone replacement^†^, n (%)
		Day	Range					
**Total**	166	77	3–1029	66 (39.8)	69 (41.6)	31 (18.7)	43 (25.9)	31 (18.7)
**irAE subtype**								
Rash	44	46	4–706	28 (63.6)	12 (27.3)	4 (9.1)	11 (25.0)	0 (0.0)
Pruritus	13	87	24–771	9 (69.2)	4 (30.8)	0 (0.0)	0 (0.0)	0 (0.0)
Vitiligo	4	111	23–204	4 (100.0)	0 (0.0)	0 (0.0)	0 (0.0)	0 (0.0)
Colitis/diarrhea	9	87	7–697	3 (33.3)	4 (44.4)	2 (22.2)	3 (33.3)	0 (0.0)
Thyroiditis	17	60	23–65	1 (5.9)	16 (94.1)	0 (0.0)	0 (0.0)	16 (94.1)
Hypophysitis	12	266	88–1029	0 (0.0)	10 (83.3)	2 (16.7)	0 (0.0)	12 (100.0)
Type 1 diabetes	3	192	124–407	0 (0.0)	0 (0.0)	3 (100.0)	0 (0.0)	3 (100.0)
Pneumonitis	26	70	8–361	11 (42.3)	6 (23.1)	9 (34.6)	15 (57.7)	0 (0.0)
Liver dysfunction	5	40	4–113	1 (20.0)	0 (0.0)	4 (80.0)	2 (40.0)	0 (0.0)
Renal dysfunction	3	35	17–64	1 (33.3)	1 (33.3)	1 (33.3)	1 (33.3)	0 (0.0)
Neuropathy	2	238	3–472	0 (0.0)	2 (100.0)	0 (0.0)	1 (50.0)	0 (0.0)
RA	3	259	20–331	2 (66.7)	1 (33.3)	0 (0.0)	1 (33.3)	0 (0.0)
RS3PE syndrome	2	47	8–85	0 (0.0)	2 (100.0)	0 (0.0)	2 (100.0)	0 (0.0)
Uveitis	2	62	46–78	1 (50.0)	1 (50.0)	0 (0.0)	0 (0.0)	0 (0.0)
Myocarditis	2	127	127	0 (0.0)	1 (50.0)	1 (50.0)	0 (0.0)	0 (0.0)
Amylase/lipase increase	2	91	60–122	0 (0.0)	2 (100.0)	0 (0.0)	0 (0.0)	0 (0.0)
Others	17	85	9–281	5 (29.4)	7 (41.2)	5 (29.4)	7 (41.2)	0 (0.0)

*irAE* immune-related adverse events, *RA* rheumatoid arthritis, *RS3PE* remitting seronegative symmetrical synovitis with pitting edema.

*Corticosteroid includes prednisolone, methylprednisolone, and dexamethasone treatment.

^†^Hormone replacements includes hydrocortisone, levothyroxine, and insulin treatment.

### NLR at onset of immune-related adverse events

**Figure 1:**
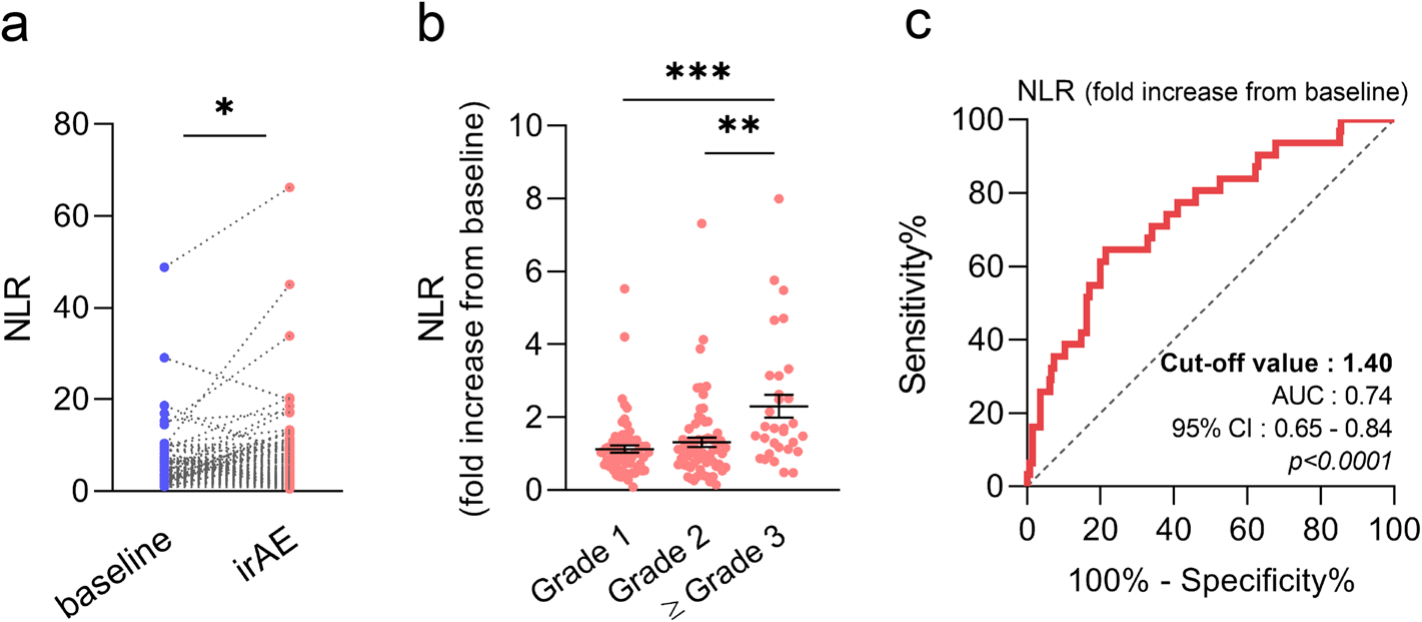
NLR trends during developing irAEs and relevance with its severity. (**a**) NLR trend from the pre-treatment until irAE occurrence (n = 166). The NLR was significantly elevated at the time the irAE occurred. (**b**) Fold increase in NLR from baseline evaluated according to the severity of irAE. Grade 3 or higher irAE showed significant elevation compared to that of other irAEs (Grade 1, n = 66; Grade 2, n = 69; Grade 3 or higher, n = 31). (**c**) ROC curve analysis of the sensitivity and specificity of NLR elevation from baseline to distinguish between Grades 1 or 2 and Grade 3 or higher irAEs. Results of ROC analysis: sensitivity 64.5%, specificity 78.5%, cut-off 1.40, and *p* < 0.0001. Statistical analysis was performed using Wilcoxon matched-pairs test (**a**) and one-way ANOVA with Tukey’s post hoc test (**b**). Data are shown as the mean ± SEM (**b**), *p < 0.05, **p < 0.01, and ***p < 0.005. *NLR* neutrophil-to-lymphocyte ratio, *irAE* immune-related adverse event, *ROC* receiver operating characteristics.

To clarify how the balance of neutrophils and lymphocytes may associate with the development of irAEs, we analyzed blood cell count data at pre-treatment (baseline) and on incidence of an irAE. In the 166 cases in 121 patients, we observed that the NLR was elevated at development of the irAE (Fig. 1a). As the baseline NLR differed widely among individual patients, we continuously observed the NLR relative trend (fold increase) from the baseline in each patient, and those values were used for analysis. When we classified irAEs according to their severity, the NLR was elevated in severe irAEs (Grade 3 or more) compared to that in other irAEs (Fig. 1b). ROC curve analysis indicated that the NLR was a significant practical factor in distinguishing severe irAEs (cut-off value 1.40, AUC 0.74, *p* < 0.0001) (Fig. 1c). Apart from the noted elevation in the NLR during the development of an irAE, other blood markers such as the white blood cell count (WBC), absolute neutrophil count (ANC), absolute lymphocyte count (ALC), and C-reactive protein (CRP) were also investigated (Supplementary Fig. S1). Similar to the NLR results, the WBC, ANC, and CRP levels were elevated, whereas the ALC was decreased in severe irAEs. Moreover, the ROC curve analysis revealed that none of these markers exceeded the specificity and sensitivity of the NLR in classifying severe irAEs (Supplementary Fig. S1a–d). Predicting which patients may experience irAEs before the initiation of treatment is clinically essential; thus, we performed multiple logistic regression analysis using patient background and laboratory test values, including the pre-treatment NLR. There was significant difference in the irAE incidence rate between NSCLC (52.9%) and HNC (31.7%); however, no significant risk factors were extracted other than cancer type (Supplementary Table S2).

### NLR in pneumonitis

**Figure 2:**
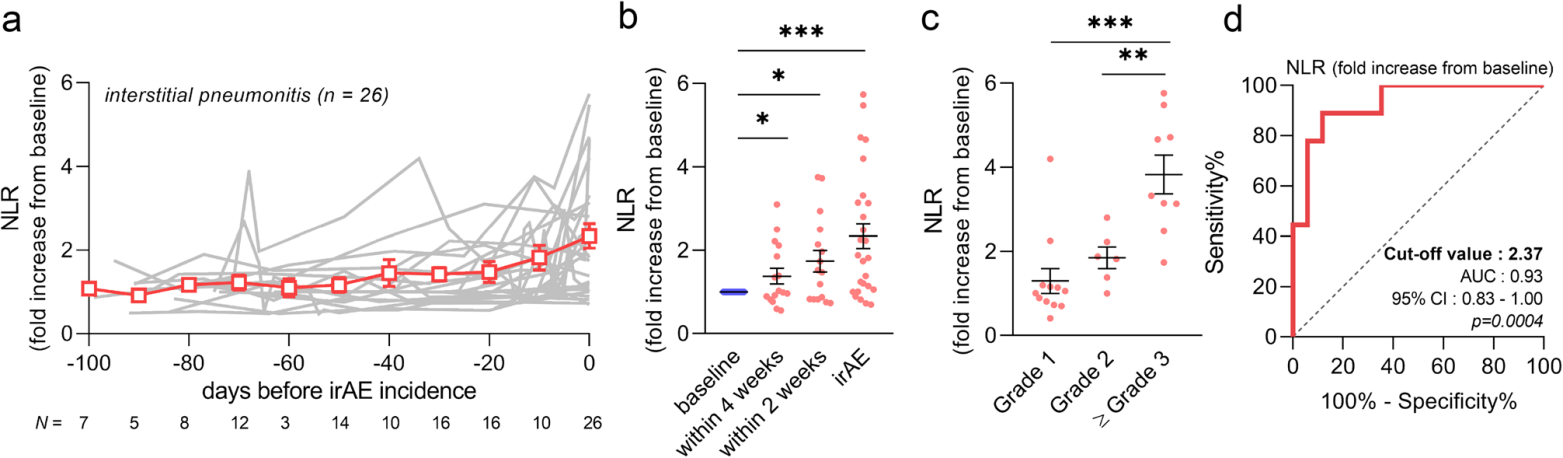
NLR trends in patients with interstitial pneumonitis. (**a**) Plotting the NLR trends during 100 days before development of ICI-related interstitial pneumonitis. The gray line indicates the individual patients (n = 26), and the red line shows the mean ± SEM. At 0 day is the initial detection of pneumonitis. (**b**) The NLR was significantly elevated not only at incidences of pneumonitis, but also 4 and 2 weeks in advance. (**c**) NLR elevation at pneumonitis development was compared among the severity of the irAEs. (**d**) ROC curve analysis of the sensitivity and specificity of NLR elevation from baseline to distinguish between Grade 1 or 2 and Grade 3 or higher pneumonitis (sensitivity 88.9%, specificity 88.2%, cut-off 2.37, *p* = 0.0004). Statistical analysis included one-way ANOVA with mixed-effects model followed by Holm-Sidak’s post hoc test (**b**), and one-way ANOVA with Tukey’s post hoc test (**c**). Data are shown as the mean ± SEM (**b**, **c**), *p < 0.05, **p < 0.01, and ***p < 0.005. *NLR* neutrophil-to-lymphocyte ratio, *ICI* immune checkpoint inhibitor, *irAE* immune-related adverse event, *ROC* receiver operating characteristics.

As pneumonitis is reported to develop at a higher rate during ICI treatment, which can be life-threatening in severe cases^35^, early prediction is essential in clinical practice. First, we investigated NLR trend over 100 days before the development of pneumonitis (n = 26). In this analysis, 0 day represents the initial detection of pneumonitis development, such as consolidation on chest X-rays. We found that the NLR gradually increased without any changes on chest X-rays or physical signs (Fig. 2a). Statistically, the NLR was already elevated approximately 4 weeks before pneumonitis appeared (Fig. 2b). Moreover, NLR elevation at the initial symptoms of pneumonitis (0 day) was well correlated with the subsequent severity (Fig. 2c). ROC curve analysis revealed that this elevation could serve as a useful biomarker to distinguish the severity of pneumonitis with high accuracy (AUC 0.93, sensitivity 88.9%, specificity 88.2%, cut-off 2.37, *p* = 0.0004) (Fig. 2d). In addition, we conducted the same analysis using Krebs von den Lungen 6 (KL-6), a conventional specific marker of interstitial pneumonitis. As expected, KL-6 was significantly increased when the pneumonitis developed. Although KL-6 was not followed up often before the irAEs occurred, a few patients who were subjected to regular measurements of KL-6 showed no elevation prior to clinical observations of pneumonitis (Supplementary Fig. S2a). Further, unlike the NLR, KL-6 elevation did not correlate with the severity of pneumonitis (AUC 0.63, sensitivity 75.0%, specificity 69.2%, cut-off 1.15, *p* = 0.3106) (Supplementary Fig. S2b,c).

### NLR in endocrine dysfunction

**Figure 3:**
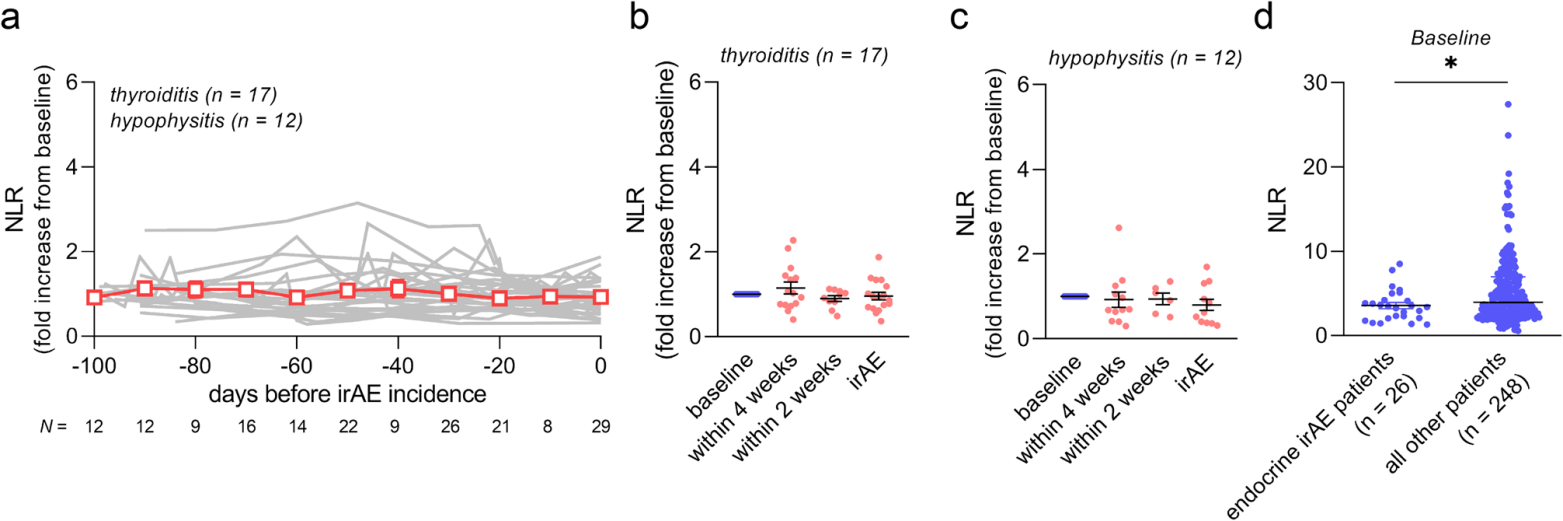
NLR trends in patients with endocrine irAEs. (**a**) Plotting the NLR trends during the 100 d before the development of ICI-related thyroiditis (n = 17) and hypophysitis (n = 12). The gray line indicates the individual patients, and the red line shows the mean ± SEM. (**b**) NLR elevation from the baseline was analyzed at each time point in thyroiditis, and (**c**) hypophysitis. (**d**) Pre-treatment NLR (baseline) was compared among patients who experienced endocrine irAEs (n = 26) and all other patients in our dataset (n = 248). Statistical analysis included one-way ANOVA with a mixed-effects model followed by Holm–Sidak’s post hoc test (**b**), one-way ANOVA with Tukey’s post hoc test (**c**), and Mann–Whitney test (**d**). Data are shown as the mean ± SEM (**b, c**), and the median in (**d**), *p < 0.05. *NLR* neutrophil-to-lymphocyte ratio, *ICI* immune checkpoint inhibitor, *irAE* immune-related adverse event.

Next, we analyzed patients who developed endocrine irAEs including thyroiditis (n = 17) and hypophysitis (n = 12). The NLR showed no elevation both in advance and at the incidence of the irAEs (Fig. 3a–c). Moreover, the pre-treatment (baseline) NLR in patients with endocrine irAEs showed lower NLRs compared to that of all other patients (Fig. 3d).

Additionally, we evaluated the NLRs trends in other cases of irAEs. Although kidney dysfunction, type 1 diabetes mellitus, rheumatism-related irAEs, and colitis tended to have an elevated NLR at development, the increase was not significant, mainly because of the small sample size of the study (Supplementary Fig. S3a–g).

### Prognostic factors in ICI treatment

In our dataset, using univariate analysis for every cancer type and in all the patients, we confirmed that patients who developed irAEs had long progression-free survival (PFS) and OS (Supplementary Figs. S4a–h, 4a,b). Using the multivariate Cox proportional hazard analysis, the presence of an irAE was an independent prognostic factor in PFS (hazard ratio (HR) 0.41, 95% CI 0.31–0.54, *p* < 0.0001), and OS (HR 0.45, 95% CI 0.33–0.62, *p* < 0.0001) as well as ECOG PS and a low NLR (Supplementary Tables S3, S4).

**Figure 4:**
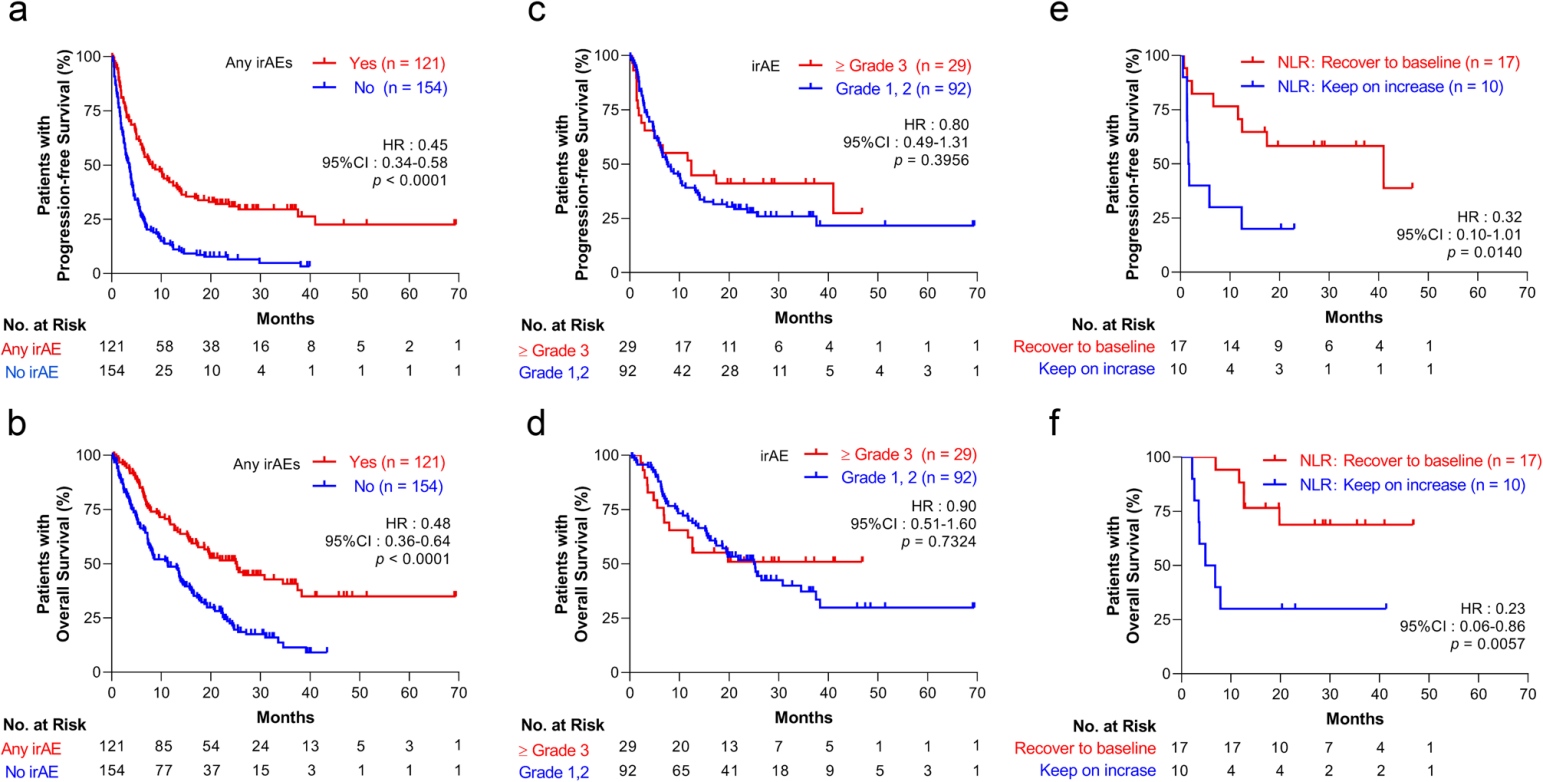
Trend in NLR estimates subsequent prognosis after severe irAE. (**a**) Kaplan-Maier analysis of PFS and (**b**) OS were performed in patients stratified according to the irAE incidence. (**c**) Among the patients with irAE development, PFS and (**d**) OS were investigated according to their irAE severity. (**e**) Among the patients who experienced Grade 3 or more severe irAEs, PFS and (**f**) OS were performed in patients stratified according to the NLR trend at 4 weeks after the development of the irAEs. Statistical analysis was performed using the log-rank test. *NLR* neutrophil-to-lymphocyte ratio, *irAE* immune-related adverse event, *PFS* progression-free survival, *OS* overall survival.

### Impact of irAE severity on prognosis in ICI therapy

As we determined that a low NLR was an independent prognostic factor, we next focused on the effect of an elevated NLR at irAE onset on survival. Since an elevated NLR was commonly observed in patients who developed a Grade 3 or higher irAE (Fig. 1b), we analyzed whether the severity of the irAE influences a patient’s survival. Patients who had a Grade 3 or higher irAE showed no significant difference in PFS and OS in every cancer types examined (Supplementary Fig. S5a–h) and in all patients (median PFS, Grade 1 or 2; 7.5 months, ≥ Grade 3; 12.4 months, HR 0.80, 95% CI 0.49–1.31; median OS, Grade 1 or 2; 25.0 months, ≥ Grade 3; not reached, HR 0.90, 95% CI 0.51–1.60) (Fig. 4c,d). In this analysis, the duration between the start of treatment and irAE development was not different in the two groups (median duration: Grade 1 or 2, 53 days; ≥ Grade 3; 89 days).

### Trend in the NLR estimated subsequent prognosis after severe irAEs

Next, we focused on the trend of elevated NLRs following Grade 3 or higher irAE development (N = 29). Patients whose NLRs could not be followed after the irAEs were excluded from this analysis (two cases). After the onset of severe irAEs, we observed two types of NLR trends. One showed that the NLR recovered to baseline levels, whereas in the second pattern, the NLR remained elevated. Therefore, we dichotomized those patients by the trend at approximately 4 weeks after the development of irAEs, and defined patients whose NLR had decreased to Grade 1 or 2 (baseline × 1.25) as the NLR recovery group (n = 17), and the other patients as the elevated NLR group (n = 10). In these two groups, patient characteristics such as age, sex, ECOG PS, primary tumor type, and administered ICI drugs exhibited no differences (Table 3). Elevated NLRs had no significant differences; however, patients maintaining high NLRs showed many incidences of pneumonitis compared to the other group. Following survival analysis, the NLR recovery group showed significantly prolonged PFS (median PFS: NLR recovery group, 41.0 months; elevated NLR group, 1.7 months; HR 0.32, 95% CI 0.10–1.01, *p* = 0.0140) and OS (median OS: NLR recovery group, not reached; elevated NLR group, 5.8 months; HR 0.23, 95% CI 0.06–0.86, *p* = 0.0057) (Fig. 4e,f). There were no differences in the duration until irAE development and the number of patients who received corticosteroid treatment after the irAE (Table 3). Thus, we identified subgroups with poor prognosis after the development of severe irAEs, and continuously following the NLR was essential to predict the prognosis of those patients.

**Table 3:** Comparison of clinical variables in patients dichotomized via NLR trend after severe irAEs.

Characteristic	NLR Recover to Baseline	NLR Elevated	*p-*value
	(n = 17), n (%)	(n = 10), n (%)	
**Age (years), median (range)**	70 (52–80)	68 (56–78)	
< 70	8 (47.1)	5 (50.0)	> 0.9999
≥ 70	9 (52.9)	5 (50.0)	
**Sex**			
Male	12 (70.6)	10 (100.0)	0.1240
Female	5 (29.4)	0 (0.0)	
**ECOG PS**			
0–1	16 (94.1)	10 (1000.0)	> 0.9999
≥ 2	1 (5.9)	0 (0.0)	
**Primary tumor type**			
NSCLC	9 (52.9)	3 (30.0)	0.4244*
Melanoma	3 (17.6)	1 (10.0)	
HNC	3 (17.6)	3 (30.0)	
RCC	2 (11.8)	3 (30.0)	
**Type of irAE incidence**			
Pneumonitis	1 (5.9)	7 (70.0)	0.0009^†^
Liver dysfunction	4 (23.5)	0 (0.0)	
Renal dysfunction	0 (0.0)	1 (10.0)	
Endocrine dysfunction	4 (23.5)	1 (10.0)	
Rash	3 (17.6)	0 (0.0)	
Colitis/diarrhea	2 (11.8)	0 (0.0)	
Other	3 (17.6)	1 (10.0)	
**Median duration until irAE incidence days (range)**	112 (6–691)	28 (2–350)	0.1512
**Corticosteroid treatment against irAE**			
Yes	11 (64.7)	8 (80.0)	0.6655
No	6 (35.3)	2 (20.0)	
**Administrated immune checkpoint inhibitors**			
Nivolumab	13 (76.5)	9 (90.0)	0.6210
Pembrolizumab	4 (23.5)	1 (10.0)	
**NLR trend (fold increase from baseline)**			
At irAE (range)	1.95 (0.47–7.99)	2.62 (1.17–4.66)	0.3343
At 4 weeks after irAE (range)	0.65 (0.28–1.23)	1.94 (1.36–4.45)	< 0.0001

*ECOG PS* Eastern Cooperative Oncology Group Performance Status, *irAE* immune-related adverse events, *NLR* neutrophil-to-lymphocyte ratio.

*NSCLC versus all other groups.

^†^Pneumonitis versus all other groups.

## Discussion

The purpose of this study was to evaluate the real-world incidence of irAEs caused by PD-1 antibody monotherapy using medical information in daily practice. This is the first study to track the NLR throughout treatment with ICIs, referring to the time of onset and severity of pneumonitis, and the feasibility of using NLR trends as a prognostic marker for patients who developed severe irAEs.

In this study, we analyzed the onset of irAEs across four cancer types: malignant melanoma, NSCLC, HNC, and RCC. Interestingly, the incidence of irAEs was high in NSCLC (52.9%) and low in HNC (31.7%). Lead time bias for the irAEs should be of concern because ICI treatment of NSCLC is often used as the front-line therapy compared to that of HNC in this cohort. To validate our findings, additional data is needed that match the treatment-line (e.g., first-line, second-line) and the observational period. However, HNC patients from our study often received radiation therapy, and many patients also had long-term lymphocytopenia. It is possible that these pre-treatments may have influenced the subsequent development of an irAE. In contrast, there was no difference in the severity of onset and clinical features of the irAEs based on the types of primary tumors. Additionally, the incidence of irAEs in our study, particularly severe adverse events classified as greater than Grade 3, was not significantly different from that reported in previous studies^4–7,15,36^.

Several studies have shown that CD8-positive lymphocytes infiltrated in tissues with irAEs and activation of lymphocytes play a central role in the development of irAEs^37–39^. Since the elevated NLR at the onset of the irAEs was accompanied with an increase in the WBC and CRP, changes in the NLR suggests irAE-induced systemic inflammation. Thus, these inflammation reactions were strongly reflected in the blood cell counts rather than in the activated immune status induced by the administration of ICIs.

We reported that the NLR trends varied according to the type of irAE; notably, pneumonitis showed a significantly elevated NLR during its development. Pulmonary toxicity is one of the most significant ICI-related toxicities. It can cause respiratory failure and death in severe cases; thus, early detection and management are crucial^35^. However, the precise mechanisms of ICI-induced pneumonitis remain unknown. Anti-PD-1 antibody is known to provoke an aberrant activation of immune cells, which attack type II alveolar epithelial cells, airway epithelial cells, and vascular endothelial cells^9^. This cytotoxicity may induce systemic inflammation and increases in the NLR. Fujisawa et al. also reported that the elevation of neutrophils and lymphopenia occurred in Grade 3 and 4 pneumonitis^40^, consistent with our results. Remarkably, we found that the NLR was gradually changed and significantly elevated before detecting the irAEs. These trends indicate that immune cells injured pulmonary tissues and triggered interstitial fibrosis, which gradually occurred before the development of specific symptoms in patients or their detection via diagnostic imaging tests. KL-6 is a specific and reliable marker for interstitial lung disease^41^; however, our results indicate that the NLR more accurately predicted the onset and severity of ICI-related pneumonitis. Previous studies showed that the increase in the serum KL-6 level depended on the type of drug-induced pneumonitis^41,42^. Specifically, diffuse alveolar damage and chronic interstitial pneumonitis showed KL-6 elevation, but bronchiolitis obliterans organizing pneumonitis, eosinophilic pneumonitis, and hypersensitivity pneumonitis did not^42^. Therefore, continuous follow-up of the NLRs combined with measurements of KL-6, diagnostic X-ray imaging, and computed tomography scanning may lead to early detection of pneumonitis and the evaluation of its severity.

In contrast, we did not observe elevated NLRs in skin-related adverse events. As most skin irAEs were Grade 1 mild rash and pruritus, we assumed that the NLR had little influence on these effects. Instead, patients who developed Grade 3 Stevens–Johnson syndrome showed a twofold increase in the NLR compared with the baseline value.

Interestingly, patients who developed thyroiditis and hypophysitis maintained a low NLR from the pre-treatment to irAE onset. The mechanism of endocrine irAEs caused by anti-PD-1 antibodies and the other factors that may contribute to individual differences remain unknown. Our results suggest that a lymphocyte-dominant immune state indicated by a low NLR may be one of the factors in the development of endocrine irAEs. Yano et al. reported that the presence of HLA-DR15 is a predictor of ICI-induced hypophysitis^23^. It is possible that endocrine irAEs may be caused by multiple triggers. Unlike pneumonitis, there were no signs of inflammation as shown by the elevated NLR or CRP levels during the developing endocrine irAE—even though the thyroid and pituitary should be attacked by activated immune cells. These results provide clues to the mechanism of endocrine irAE development, and further studies are warranted to explore them further.

IrAE incidence and maintaining low NLRs are the most established prognostic factors in treatment with ICIs^14–16,27–29^, and our data was consistent with these results. On the contrary, our results showed that the NLR was elevated at the development of Grade 3 or higher irAEs. Therefore, we analyzed how the subsequent prognosis was affected by these elevated NLR. Interestingly, patients whose NLR continued to increase or remained at high levels after irAE development showed considerably poor PFS and OS compared with NLR recovery patients. These results suggest that normalization of the NLR through therapeutic interventions may be a prognostic marker after the occurrence of severe irAEs. Patients with continuously elevated NLR were more likely to have pneumonitis. The association among the types of irAEs, the treatment given for them, and the subsequent prognosis will need further study. The precise mechanism that mediates increasing NLRs that correspond to an ICI-related poor prognosis remains unknown, and further research is needed to validate our results before putting them in clinical practices. However, if validated, following the trends in the NLRs may help to predict the prognosis of patients and influence their clinical treatment.

There were several limitations to this study, mainly because this was a single-center, retrospective analysis. In this study, we could not investigate NLR trends in irAEs other than pneumonitis and endocrinopathies due to the small sample size. Future work should investigate and analyze additional data on those irAEs to fully understand the association between irAEs and NLRs. The prognostic impact of the NLR trend after the irAE occurrence was analyzed across cancer types. Although there was no significant difference in the type of cancer among the groups, future work will focus on validating these observations for each type of cancer. We could not detect the risk factors for developing irAEs at the pre-treatment. Pavan et al*.* reported that a low platelet to lymphocyte ratio (PLR) was a risk factor for irAEs using multivariate analysis in advanced NSCLC^43^; however, our datasets did not reproduce these results (Supplementary Table S1). This discrepancy may be related to our patient background, as we included patients across various cancer types. However, unlike adverse effects with conventional chemotherapy, irAEs can develop months or years after the first administration. It may be difficult to predict the risk of irAEs from the immune status before treatment alone; thus, studies continuously monitoring immune conditions during treatment are needed.

Finally, the prediction of irAEs using routinely available tests has great clinical value. We currently regularly conduct many laboratory tests and diagnostic imaging for the early detection of irAEs. If our results are further validated, we may screen cases of irAEs using the NLR before conducting diagnostic organ-specific tests or imaging tests. This will help reduce the patient burden, decrease sample collection, and improve the healthcare economy. As ICIs have broad indications, many patients may be treated with ICIs^44^. Our results will contribute to promote the proper management of ICIs and help guarantee patient safety.

In conclusion, we found that the elevation of the NLR was correlated with the severity of irAEs. In particular, following the NLR trend could help predict the onset of pneumonitis and the patient’s prognosis after the occurrence of a severe irAE. Although further research is needed on irAEs in other organs, continuous monitoring of NLR trends and an understanding of its characteristics has the potential to be useful in estimating irAE onset, severity, and subsequent prognosis of patients undergoing ICI-based therapy.

## Supplementary Information

Supplementary Information.

## Data Availability

The data that support the findings of this study are available from the corresponding author upon reasonable request.
