# Supplementary material for: Continuous monitoring of neutrophils to lymphocytes ratio for estimating the onset, severity, and subsequent prognosis of immune related adverse events
Source: Sci Rep. 2021 Jan 14;11:1324. doi: 10.1038/s41598-020-79397-6 (PMC7809015; doi:10.1038/s41598-020-79397-6)
Supplement: Supplementary file 1 — Supplementary Information. [file 41598_2020_79397_MOESM1_ESM.docx]

Supplementary Material

Continuous monitoring of neutrophils to lymphocytes ratio for estimating the onset, severity, and subsequent prognosis of immune related adverse events

**Ryosuke Matsukane^1,2^, Hiroyuki Watanabe^1^, Haruna Minami^1^, Kojiro Hata^1,2^, Kimitaka Suetsugu^1^, Toshikazu Tsuji^1^, Satohiro Masuda^1,2,3^, Isamu Okamoto^4^, Takashi Nakagawa^5^, Takamichi Ito^6^, Masatoshi Eto^7^, Masaki Mori^8^, Yoichi Nakanishi^4^, and Nobuaki Egashira^1,2*^**

^1^Department of Pharmacy, Kyushu University Hospital, Fukuoka, Japan

^2^Department of Clinical Pharmacology and Biopharmaceutics, Graduate School of Pharmaceutical Sciences, Kyushu University, Fukuoka, Japan

^3^Department of Clinical Pharmacy, Faculty of Pharmaceutical Sciences, International University of Health and Welfare Narita Hospital, Chiba, Japan

^4^Research Institute for Diseases of the Chest, Graduate School of Medical Sciences, Kyushu University, Fukuoka, Japan

^5^Department of Otorhinolaryngology, Graduate School of Medical Sciences, Kyushu University, Fukuoka, Japan

^6^Department of Dermatology, Graduate School of Medical Sciences, Kyushu University, Fukuoka, Japan

^7^Department of Urology, Graduate School of Medical Sciences, Kyushu University, Fukuoka, Japan

^8^Department of Surgery and Science, Graduate School of Medical Sciences, Kyushu University, Fukuoka, Japan

*Correspondence to:

Nobuaki Egashira, PhD

Department of Pharmacy, Kyushu University Hospital

Department of Clinical Pharmacology and Biopharmaceutics, Graduate School of Pharmaceutical Sciences, Kyushu University

3-1-1 Maidashi, Higashi-ku, Fukuoka, 812-8582, Japan

TEL: +81-92-642-5920, FAX: +81-92-642-5937

Email: [n-egashi@pharm.med.kyushu-u.ac.jp](mailto:n-egashi@pharm.med.kyushu-u.ac.jp)

**Supplementary Table 1.**

**Multivariate logistic analysis of risk factors associated with irAE incidences.**

|  | Total | Melanoma | | NSCLC | | HNC | | RCC | |
| --- | --- | --- | --- | --- | --- | --- | --- | --- | --- |
|  | n | n, (%) | | n, (%) | | n, (%) | | n, (%) | |
| Total patients | 275 | 55 |  | 119 |  | 63 |  | 38 |  |
| Patients with irAE | 121 | 23 | (41.8) | 63 | (52.9) | 20 | (31.7) | 15 | (39.5) |
| Number of irAEs |  |  |  |  |  |  |  |  |  |
| 1 | 86 | 16 | (29.1) | 46 | (38.7) | 15 | (23.8) | 9 | (23.7) |
| 2 | 26 | 3 | (5.5) | 15 | (12.6) | 4 | (6.3) | 4 | (10.5) |
| ≥3 | 9 | 4 | (7.3) | 2 | (1.7) | 1 | (1.6) | 2 | (5.3) |
|  |  | G1–2 | ≥G3 | G1–2 | ≥G3 | G1–2 | ≥G3 | G1–2 | ≥G3 |
| Total | 166 | 30 | 4 | 69 | 14 | 19 | 7 | 17 | 6 |
| irAE subtype |  |  |  |  |  |  |  |  |  |
| Rash | 44 | 8 | 0 | 22 | 3 | 5 | 0 | 5 | 1 |
| Pruritus | 13 | 4 | 0 | 5 | 0 | 2 | 0 | 2 | 0 |
| Vitiligo | 4 | 4 | 0 | 0 | 0 | 0 | 0 | 0 | 0 |
| Colitis / diarrhea | 9 | 1 | 0 | 4 | 2 | 1 | 0 | 1 | 0 |
| Thyroiditis | 17 | 3 | 0 | 7 | 0 | 6 | 0 | 1 | 0 |
| Hypophysitis | 12 | 2 | 0 | 5 | 0 | 1 | 1 | 2 | 1 |
| Type 1 diabetes | 3 | 0 | 3 | 0 | 0 | 0 | 0 | 0 | 0 |
| Pneumonitis | 26 | 1 | 0 | 12 | 5 | 1 | 3 | 3 | 1 |
| Liver dysfunction | 5 | 0 | 0 | 1 | 1 | 0 | 2 | 0 | 1 |
| Renal dysfunction | 3 | 0 | 0 | 1 | 0 | 1 | 0 | 0 | 1 |
| Neuropathy | 2 | 1 | 0 | 1 | 0 | 0 | 0 | 0 | 0 |
| Rheumatoid arthritis | 3 | 0 | 0 | 2 | 0 | 1 | 0 | 0 | 0 |
| RS3PE syndrome | 2 | 1 | 0 | 1 | 0 | 0 | 0 | 0 | 0 |
| Uveitis | 2 | 1 | 0 | 1 | 0 | 0 | 0 | 0 | 0 |
| Myocarditis | 2 | 0 | 0 | 0 | 0 | 0 | 0 | 1 | 1 |
| Amylase/Lipase increase | 2 | 0 | 0 | 1 | 0 | 0 | 0 | 1 | 0 |
| Others | 17 | 4 | 1 | 6 | 3 | 1 | 1 | 1 | 0 |

NSCLC, non-small cell lung cancer; HNC, head and neck cancer; RCC, renal cell carcinoma; irAE, immune-related adverse event; RS3PE, remitting seronegative symmetrical synovitis with pitting edema

**Supplementary Table 2.**

**Multivariate logistic analysis of risk factors associated with irAE incidences.**

|  | n  (% of total) | All Grades | | |  | ≧Grade 3 | | |
| --- | --- | --- | --- | --- | --- | --- | --- | --- |
| Variable |  | OR | 95% CI | *p*-values |  | OR | 95% CI | *p*-values |
| Age, years (≦65) | 102 (37.1%) | 1.17 | 0.70–1.97 | 0.552 |  | 0.96 | 0.42–2.19 | 0.913 |
| Tumor type |  |  |  | 0.025 |  |  |  | 0.868 |
| NSCLC vs HNC |  | 2.78 | 1.35–5.73 | 0.006 |  | 0.97 | 0.32–2.95 | 0.961 |
| Sex, Female | 72 (26.2%) | 0.80 | 0.43–1.48 | 0.475 |  | 0.48 | 0.16–1.42 | 0.186 |
| ECOG PS (0–1) | 251 (91.3%) | 1.94 | 0.74–5.06 | 0.176 |  | 2.71 | 0.34–21.80 | 0.346 |
| No. of treatment (0–1) | 181 (65.8%) | 1.32 | 0.76–2.30 | 0.326 |  | 0.98 | 0.40–2.35 | 0.956 |
| Body Mass Index (>20.0) | 175 (61.8%) | 0.84 | 0.48–1.49 | 0.549 |  | 1.05 | 0.41–2.65 | 0.921 |
| Laboratory data |  |  |  |  |  |  |  |  |
| L-WBC (<6.0x10^3^) | 139 (50.5%) | 1.19 | 0.68–2.09 | 0.533 |  | 2.12 | 0.85–5.26 | 0.105 |
| L-NLR (<3.80) | 137 (49.8%) | 1.18 | 0.67–2.06 | 0.563 |  | 1.62 | 0.65–4.02 | 0.300 |
| L-PLR (<240.0) | 136 (49.5%) | 1.16 | 0.66–2.04 | 0.611 |  | 0.86 | 0.34–2.04 | 0.683 |

Statistical analysis was performed using the multiple logistic regression analysis.

OR, odds ratio; CI, confidence interval; irAE, immune-related adverse event; NSCLC, non-small cell lung cancer; HNC, head and neck cancer; ECOG PS, Eastern Cooperative Oncology Group Performance Status; L-WBC, white blood cell lower than the median; L-NLR, neutrophil-to-lymphocyte ratio lower than the median; L-PLR, platelet-to- lymphocyte ratio lower than the median.

**Supplementary Table 3**.

**Univariate and multivariate analysis of clinical variables associated with progression free survival.**

|  |  | Progression Free Survival | | | |
| --- | --- | --- | --- | --- | --- |
|  | n (% of total) | Univariate | | Multivariate | |
| Variable |  | HR (95% CI) | *p*-value | HR (95% CI) | *p*-value |
| Age, ≦65 | 102 (37.1%) | 0.88 (0.68–1.16) | 0.380 |  |  |
| Primary tumour^#^ | - | - | - | - | 0.0507^#^ |
| Sex |  |  |  |  |  |
| Female | 72 (26.2%) | 0.94 (0.70–1.25) | 0.653 |  |  |
| ECOG PS |  |  |  |  |  |
| 0–1 | 251 (91.3%) | 0.28 (0.13–0.59) | <0.0001 | 0.25 (0.16–0.40) | <0.0001 |
| No. of treatment |  |  |  |  |  |
| 0–1 | 181 (65.8%) | 0.85 (0.65–1.12) | 0.232 |  |  |
| No. of metastasis |  |  |  |  |  |
| 0–1 | 127 (46.2%) | 0.84 (0.65–1.09) | 0.197 |  |  |
| Body Mass Index |  |  |  |  |  |
| >20.0 | 178 (64.7%) | 0.81 (0.62–1.07) | 0.120 |  |  |
| irAEs |  |  |  |  |  |
| Present | 121 (44.0%) | 0.44 (0.34–0.57) | <0.0001 | 0.41 (0.31–0.54) | <0.0001 |
| Laboratory data (Pre-treatment) |  |  |  |  |  |
| L-NLR (<3.80) | 137 (49.8%) | 0.58 (0.44–0.75) | <0.0001 | 0.59 (0.44–0.79) | 0.0003 |

HR, hazard ratio; CI, confidence interval; ECOG PS, Eastern Cooperative Oncology Group Performance Status; irAE, immune-related adverse event; L-NLR, neutrophil-to-lymphocyte ratio lower than the median; PFS, progression-free survival; NSCLS, non-small cell lung cancer; HNC, head and neck cancer; RCC, renal cell cancer.

^#^ PFS (HR, 95% CI, *p*): NSCLC vs. HNC (1.46, 1.03–2.06, 0.0340), NSCLC vs. RCC (1.65, 1.06–2.57, 0.0261).

**Supplementary Table 4.**

**Univariate and multivariate analysis of clinical variables associated with overall survival.**

|  |  | Overall Survival | | | |
| --- | --- | --- | --- | --- | --- |
|  | n (% of total) | Univariate | | Multivariate | |
| Variable |  | HR (95% CI) | *p*-value | HR (95% CI) | *p*–value |
| Age, ≦65 | 102 (37.1%) | 0.78 (0.58–1.05) | 0.107 |  |  |
| Primary tumour^#^ | - | - | - | - | 0.0411^#^ |
| Sex |  |  |  |  |  |
| Female | 72 (26.2%) | 0.87 (0.64–1.19) | 0.399 |  |  |
| ECOG PS |  |  |  |  |  |
| 0–1 | 251 (91.3%) | 0.23 (0.10–0.54) | <0.0001 | 0.18 (0.11–0.29) | <0.0001 |
| No. of treatment |  |  |  |  |  |
| 0–1 | 181 (65.8%) | 0.86 (0.64–1.18) | 0.340 |  |  |
| No. of metastasis |  |  |  |  |  |
| 0–1 | 127 (46.2%) | 0.84 (0.67–1.19) | 0.439 |  |  |
| Body Mass Index |  |  |  |  |  |
| >20.0 | 178 (64.7%) | 0.65 (0.48–0.89) | 0.0040 | 0.76 (0.0.55–1.05) | 0.0956 |
| irAEs |  |  |  |  |  |
| Present | 121 (44.0%) | 0.48 (0.36–0.64) | <0.0001 | 0.45 (0.33–0.62) | <0.0001 |
| Laboratory data (Pre-treatment) |  |  |  |  |  |
| L-NLR (<3.80) | 137 (49.8%) | 0.47 (0.35–0.64) | <0.0001 | 0.52 (0.38–0.72) | <0.0001 |

HR, hazard ratio; CI, confidence interval; ECOG PS, Eastern Cooperative Oncology Group Performance Status; irAE, immune-related adverse event; L-NLR, neutrophil-to-lymphocyte ratio lower than the median; PFS, progression-free survival; NSCLS, non-small cell lung cancer; HNC, head and neck cancer; RCC, renal cell cancer.

^#^OS (HR, 95% CI, *p*): Melanoma vs. RCC (2.35, 1.30–4.25, 0.0048), NSCLC vs. RCC (1.84, 1.06–3.20, 0.0311).


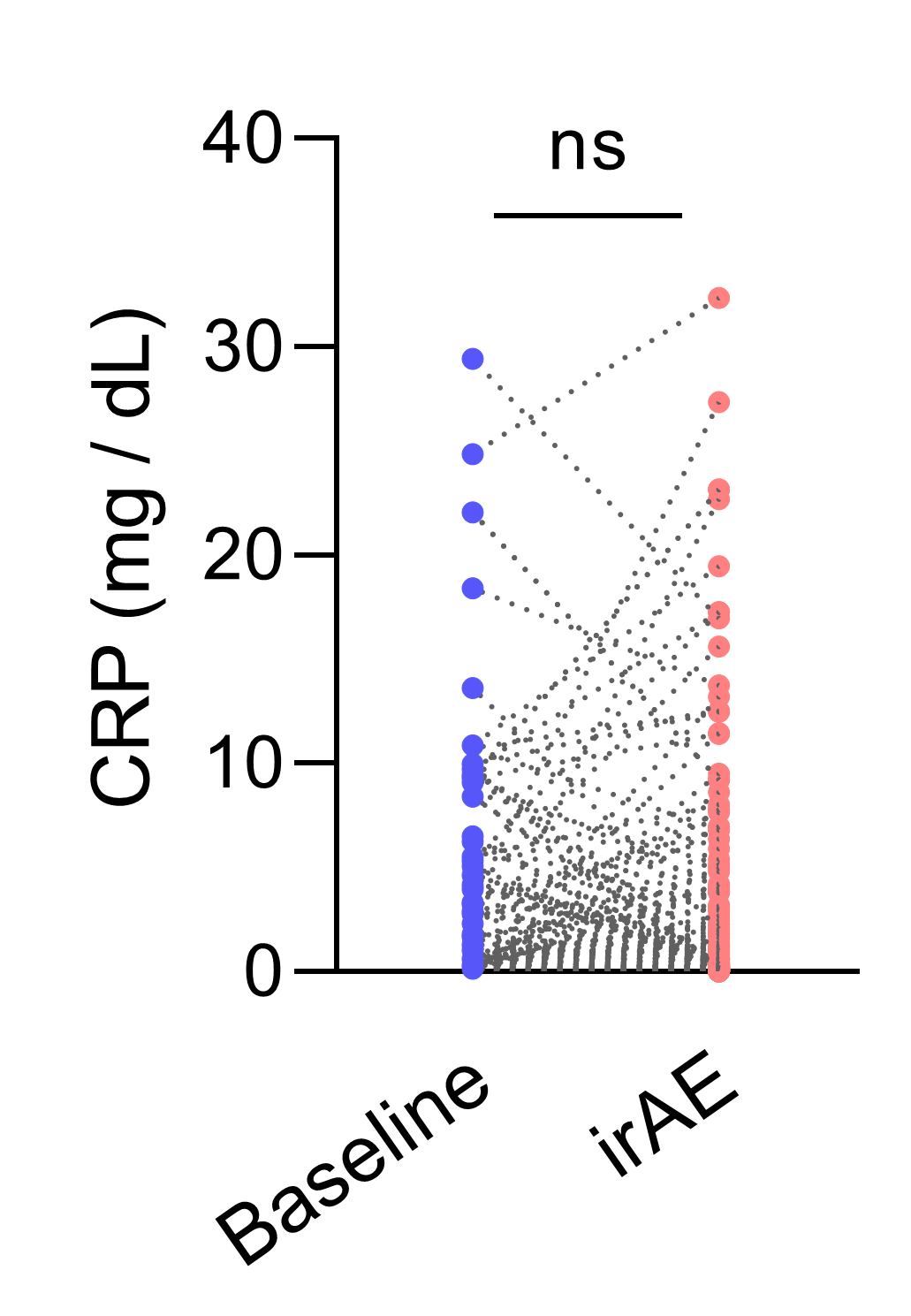

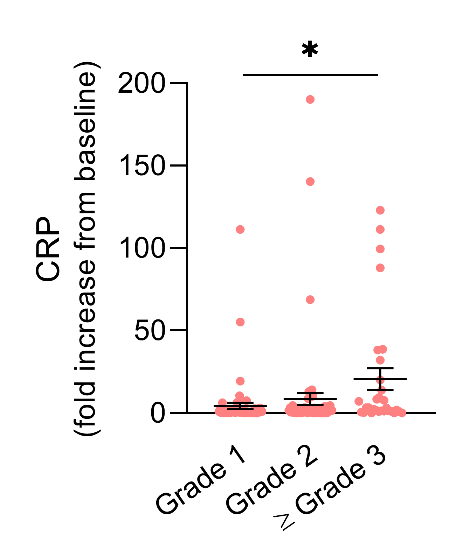

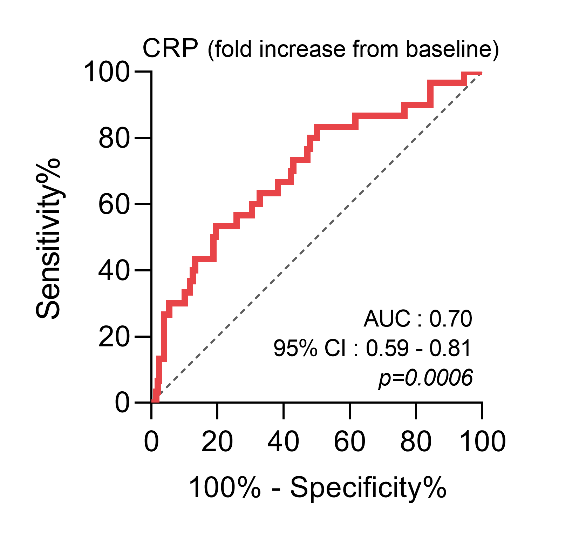

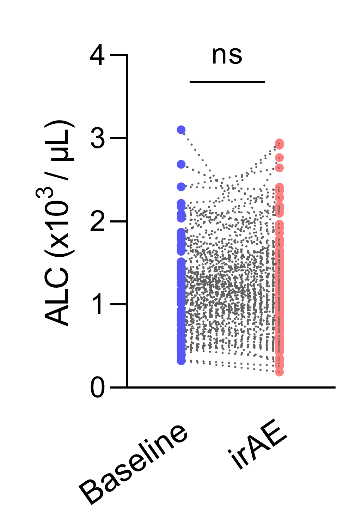

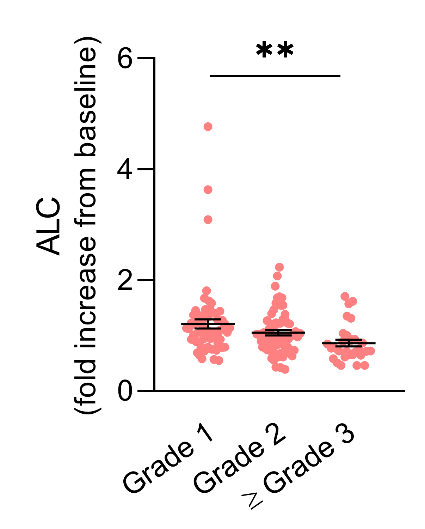

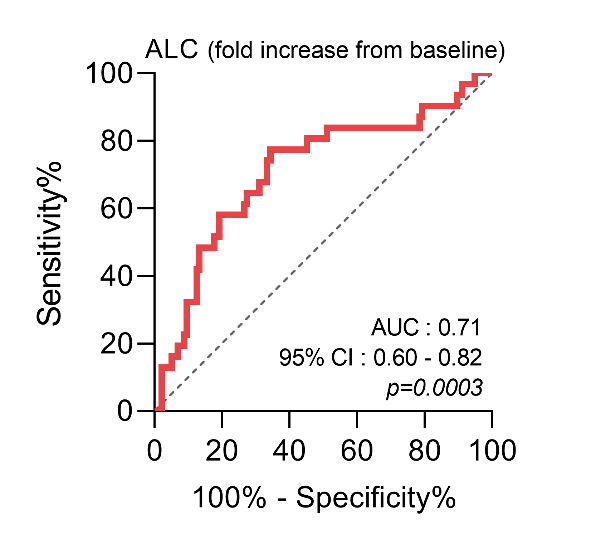

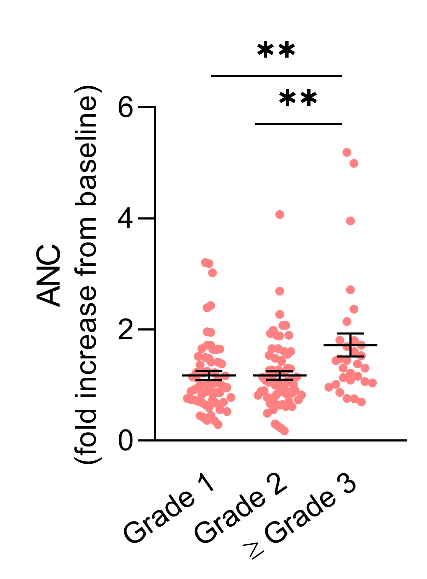

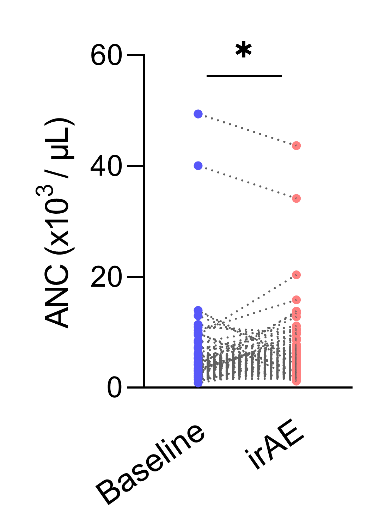

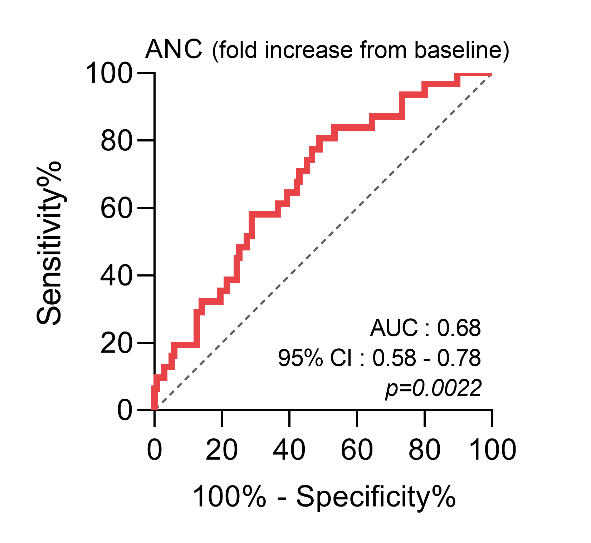

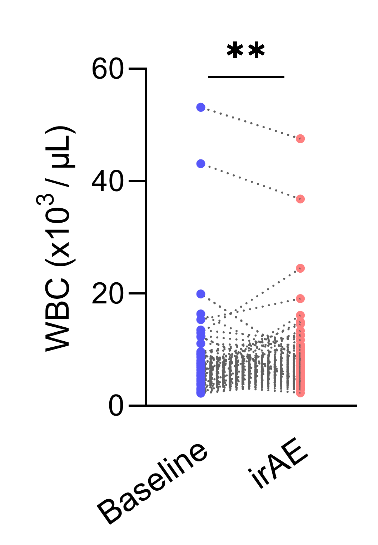

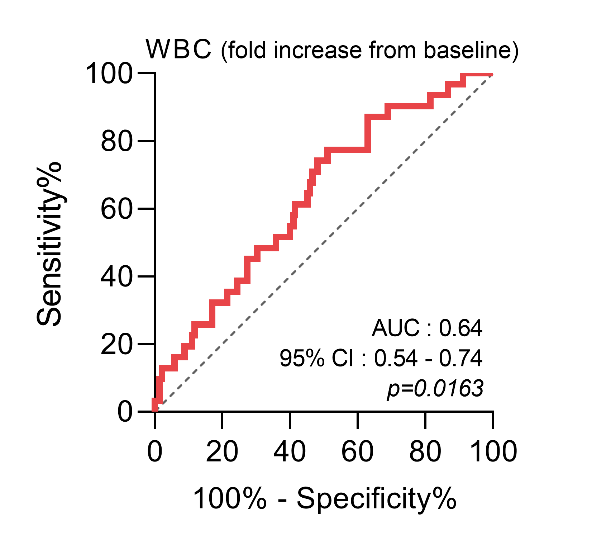

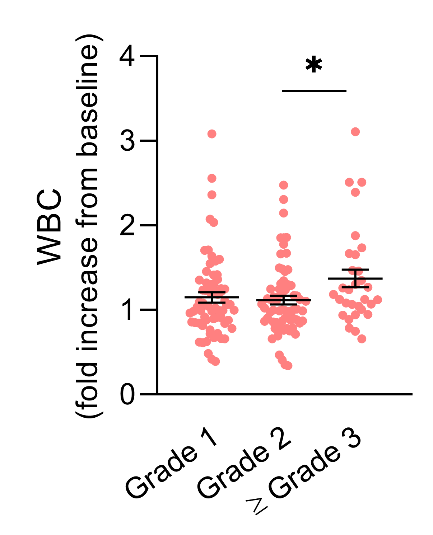


c

a

b

d

**Supplementary Fig. 1.**

**Trends of blood markers during the development of irAE and relevance with its severity.**

(**a**) WBC trends from pre-treatment to irAE occurrence (n=166). Fold-increase in the WBC from baseline was evaluated according to the severity of the irAEs. ROC curve analysis evaluated the sensitivity and specificity of WBC trends from baseline to distinguish between Grade 1 or 2 and Grade 3 or more severe irAEs. Result of ROC curve analysis: sensitivity 80.0%, specificity 46.9%, cut-off 1.00, and *p*=0.0163. (**b**) Result of ROC analysis on the absolute neutrophil count (ANC): sensitivity 83.3%, specificity 49.2%, cut-off 1.01, and *p*=0.0022. (**c**) Result of ROC curve analysis on the absolute lymphocyte count (ALC): sensitivity 76.7%, specificity 64.6%, cut-off 0.94, and *p*=0.0003. (**d**) Result of ROC curve analysis on c-reactive protein (CRP): sensitivity 86.2%, specificity 49.6%, cut-off 0.86, and *p*=0.0006. Statistical analysis included Wilcoxon matched-pairs test and one-way ANOVA with Tukey’s post hoc test. Data are shown as the mean ± SEM, **p*<0.05 and ***p*<0.01. irAE, immune-related adverse event; WBC, white blood cell count; ROC, receiver operating characteristics.


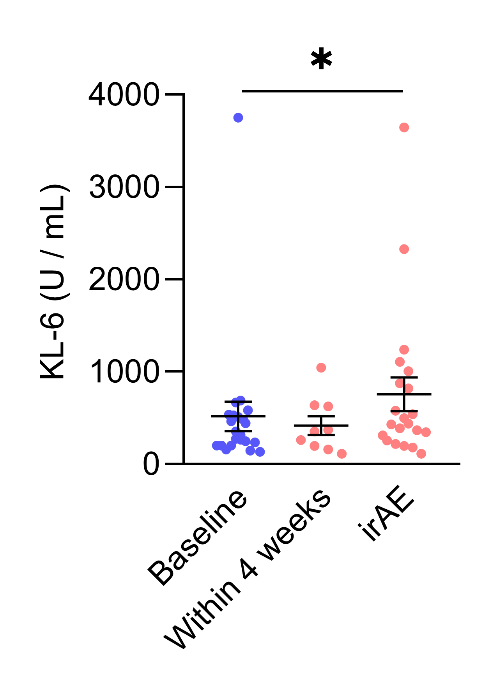


b

a


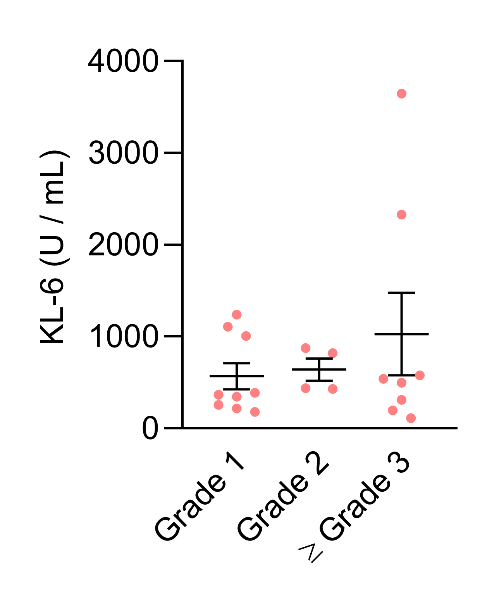


c


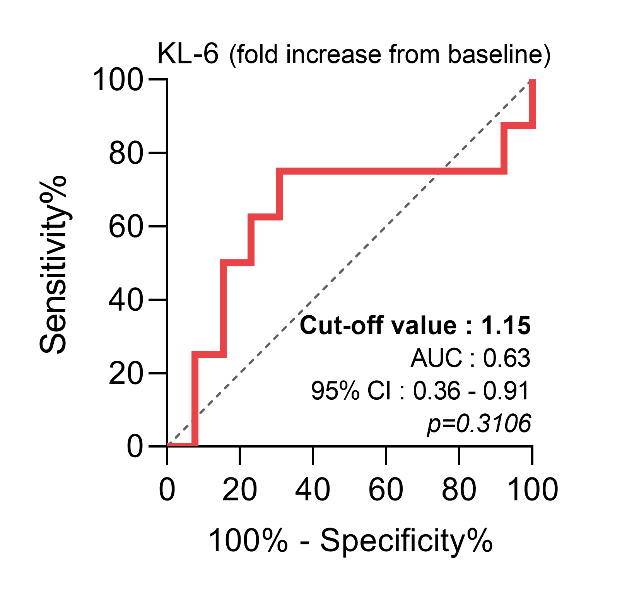


**Supplementary Fig. 2**.

**KL-6 trends in patients with interstitial pneumonitis.**

(**a**) KL-6 was significantly elevated at pneumonitis development but not in advance. (**b**) KL-6 elevation at interstitial pneumonitis development was compared among the severe irAEs. (**c**) ROC curve analysis of the sensitivity and specificity of KL-6 elevation from baseline to distinguish between Grade 1 or 2 and Grade 3 or more severe interstitial pneumonitis (sensitivity 75.0%, specificity 69.2%, cut-off 1.15, *p*=0.3106). Statistical analysis included one-way ANOVA with mixed-effects model followed by Holm-Sidak’s post hoc test (**a**) and one-way ANOVA with Tukey’s post hoc test (**b**). Data are shown as the mean ± SEM (**a**, **b**), **p*<0.05. KL-6, Krebs von den Lungen 6; irAE, immune-related adverse event.


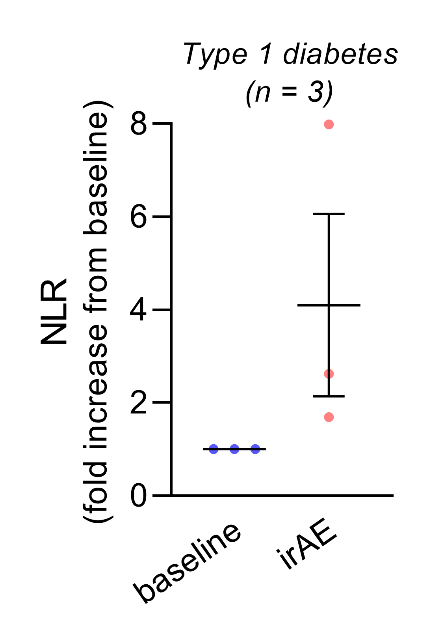

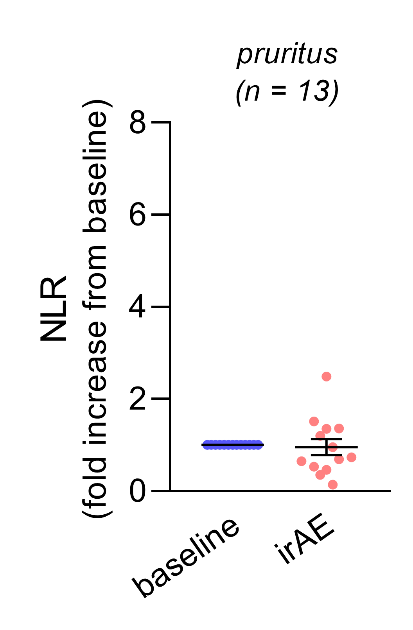


e

a


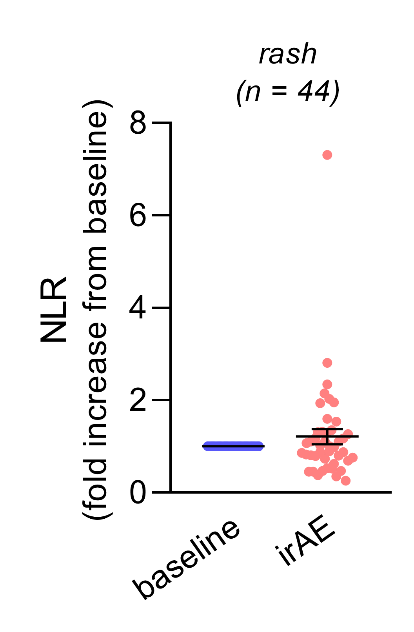

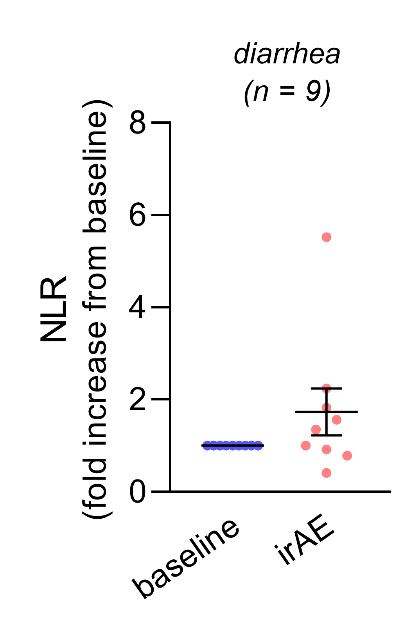

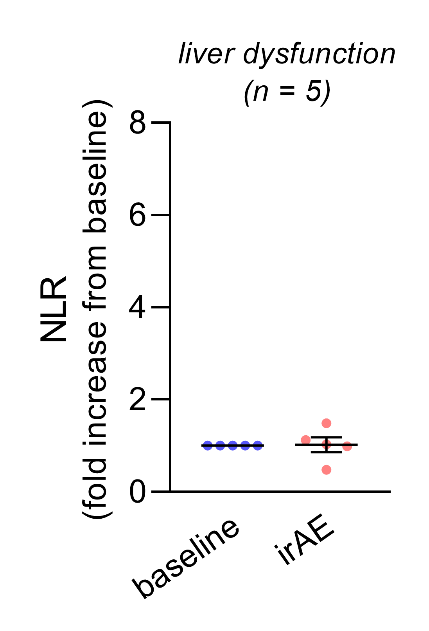

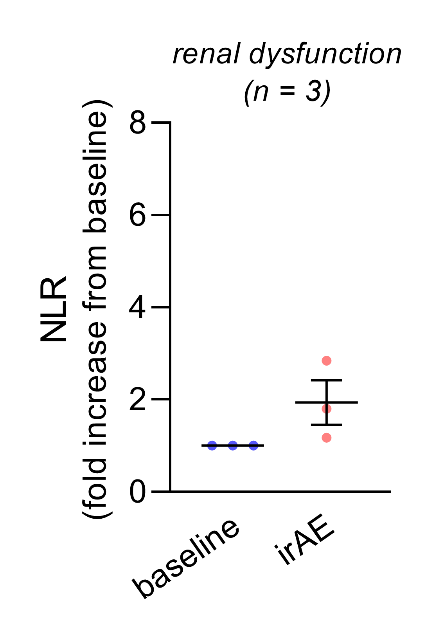

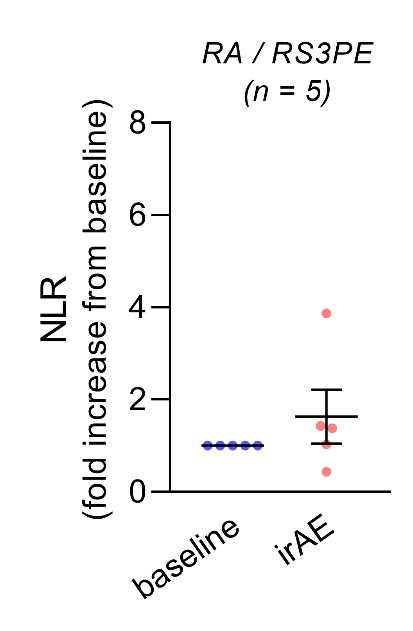


b

g

c

d

f

**Supplementary Fig. 3.**

**NLR trends at various incidences of irAEs.**

(**a**) Rash, (**b**) pruritus, (**c**) diarrhea, (**d**) liver dysfunction, (**e**) rheumatoid arthritis (RA) and remitting seronegative symmetrical synovitis with pitting edema (RS3PE), (**f**) renal dysfunction, and (**g**) type 1 diabetes mellitus. Statistical analysis was performed using the paired *t*-test. Data are shown as the mean ± SEM. NLR, neutrophil-to-lymphocyte ratio; irAE, immune-related adverse event.


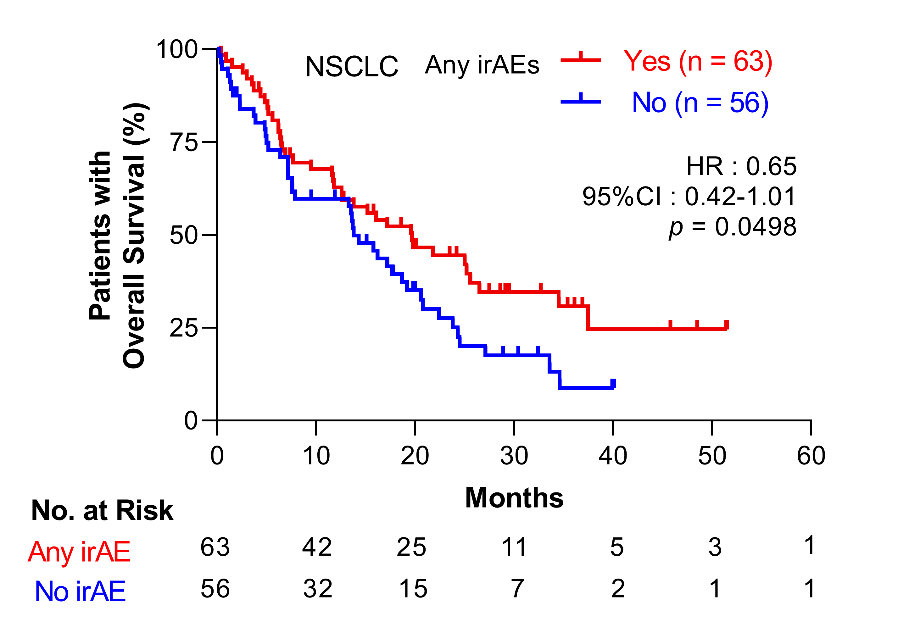

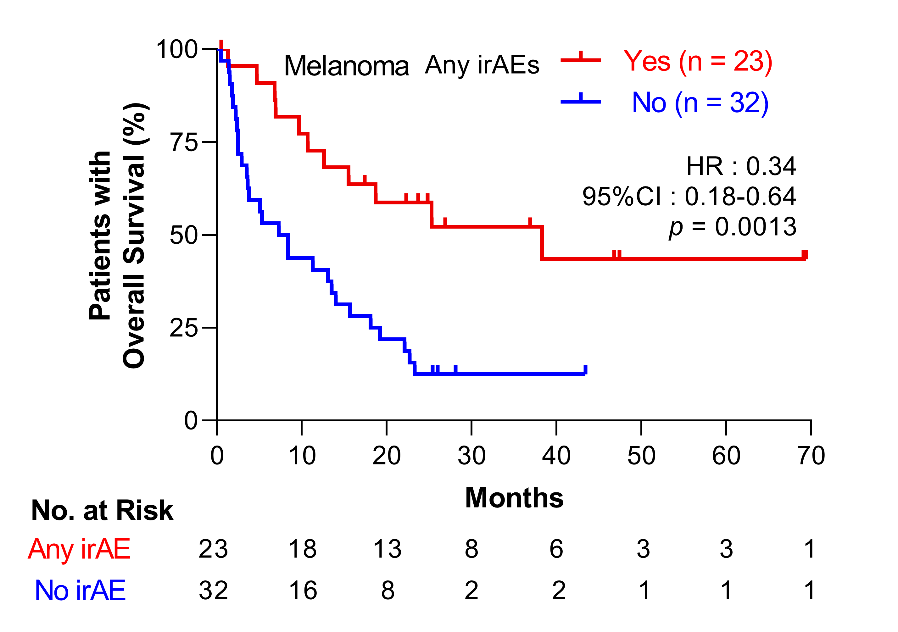


a

b


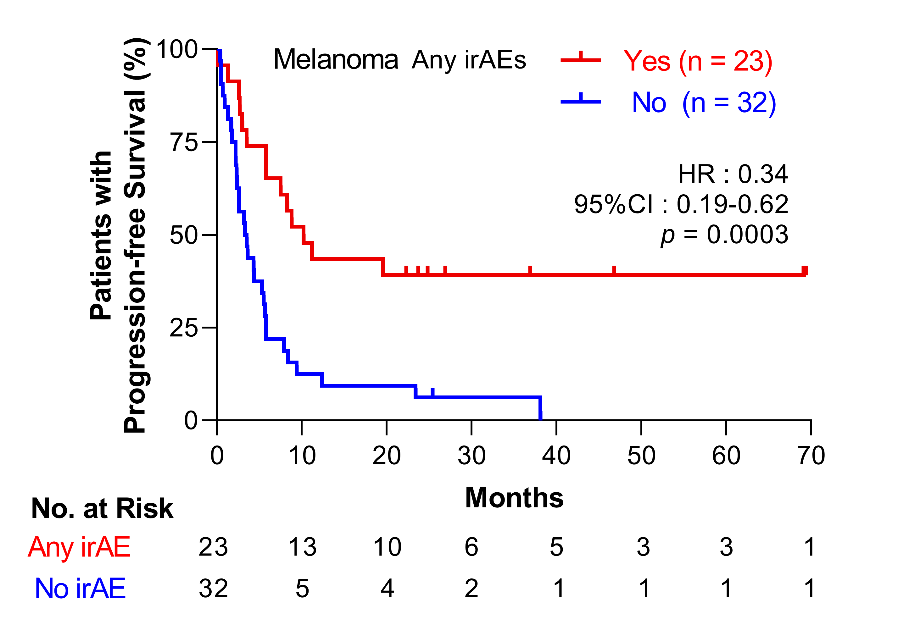

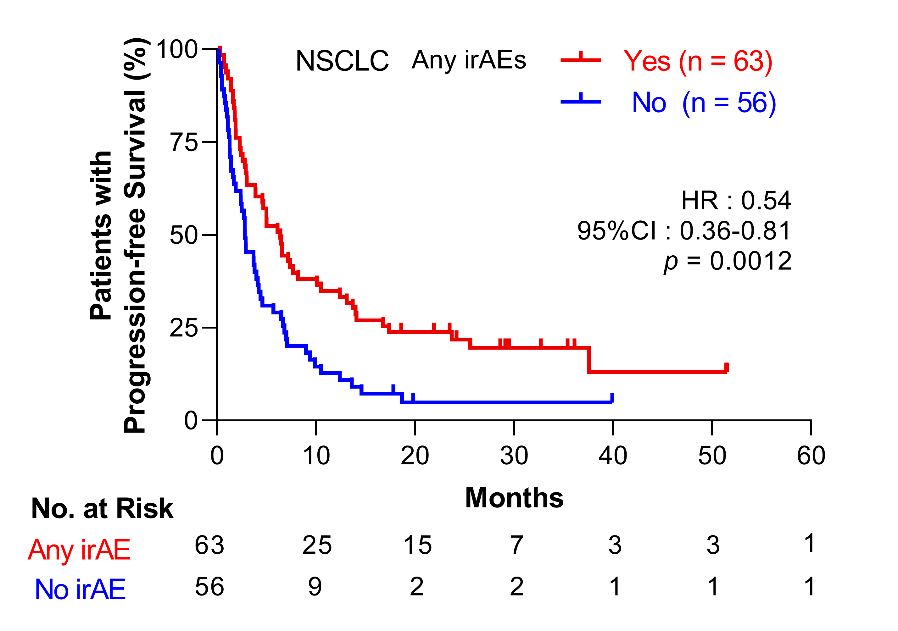

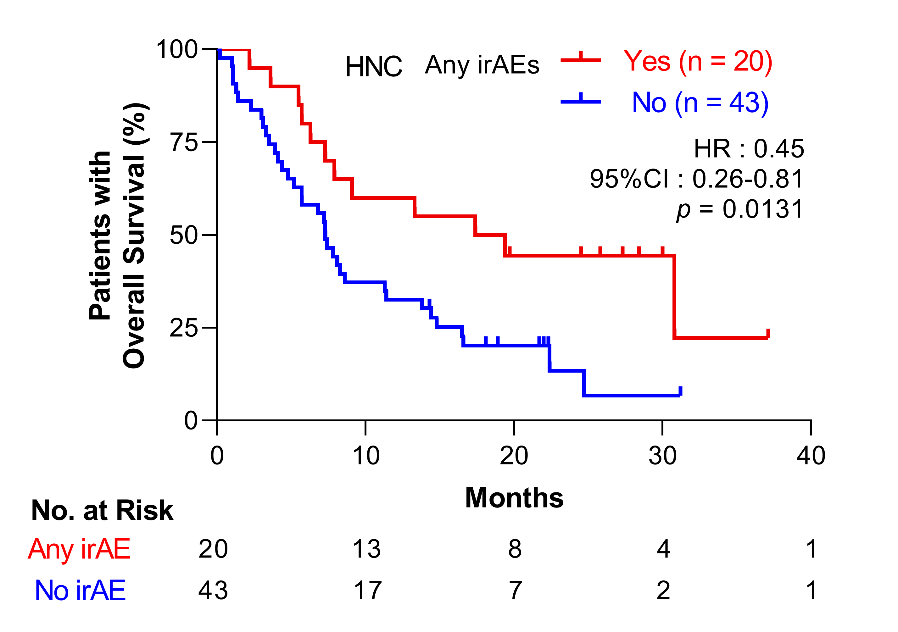

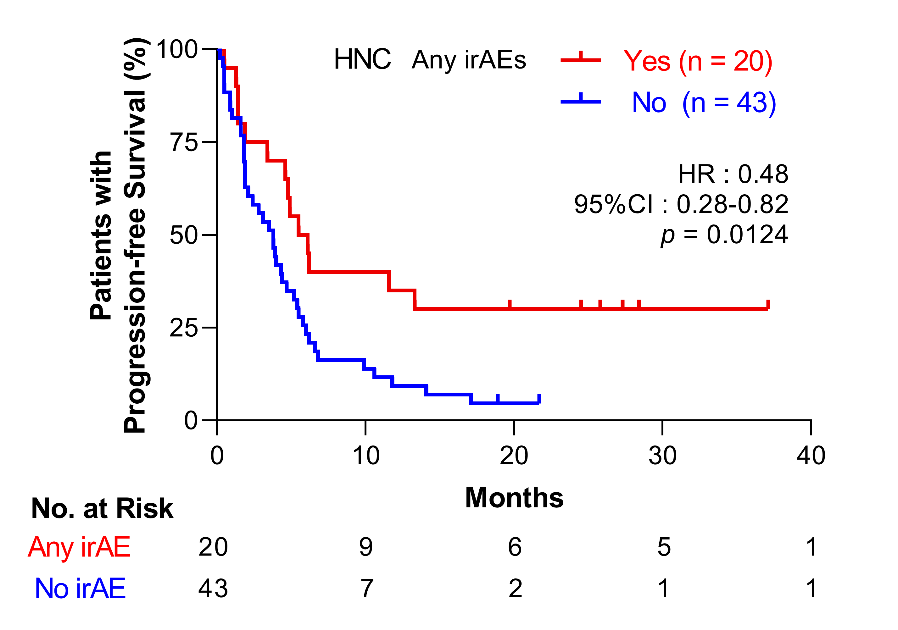

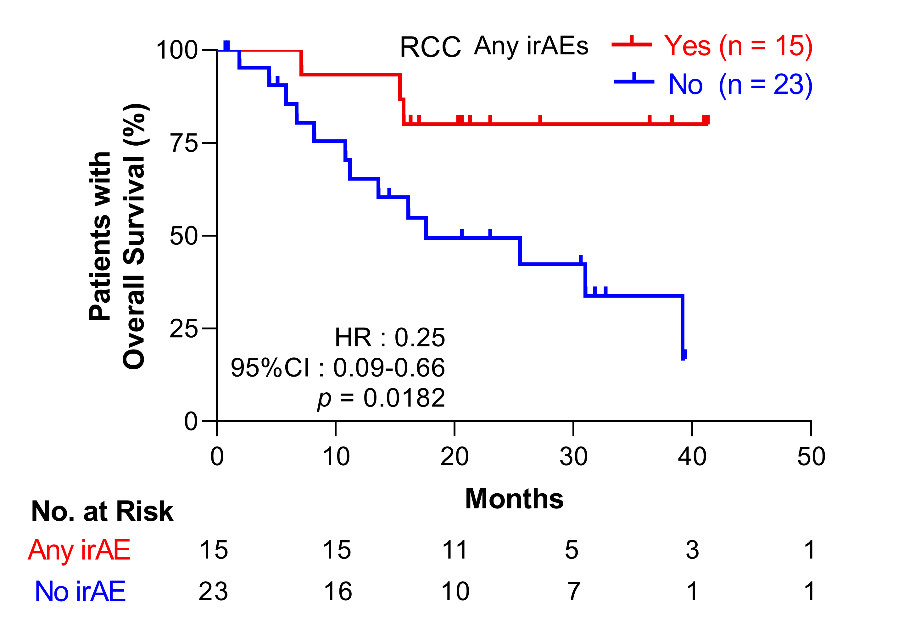

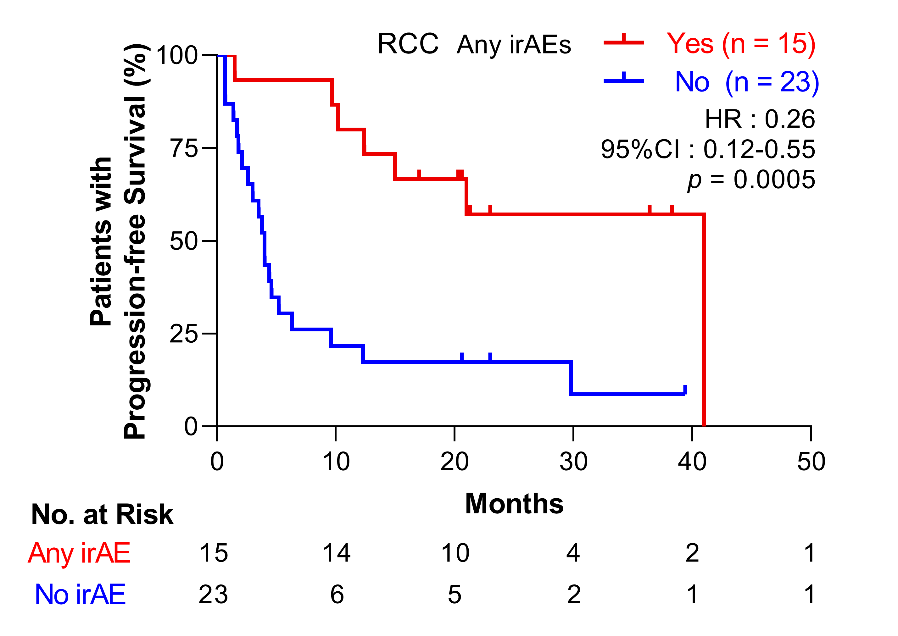


c

d

e

f

g

h

**Supplementary Fig. 4.**

**Kaplan-Meier survival analysis of PFS and OS according to the presence of an irAE.**

(**a**) PFS in melanoma patients; median PFS: no irAEs 3.4 months, any irAEs 10.2 months. (**b**) OS in melanoma patients; median OS: no irAEs 7.9 months, any irAEs 38.3 months. (**c**) PFS in non-small cell lung cancer (NSCLC) patients; median PFS: no irAEs 2.8 months, any irAEs 6.4 months. (**d**) OS in NSCLC patients; median OS: no irAEs 13.8 months, any irAEs 19.7 months. (**e**) PFS in head and neck cancer (HNC) patients; median PFS: no irAEs 3.8 months, any irAEs 5.8 months. (**f**) OS in HNC patients; median OS: no irAEs 7.3 months, any irAEs 18.4 months. (**g**) PFS in renal cell carcinoma (RCC) patients; median PFS: no irAEs 4.0 months, any irAEs 41.0 months. (**h**) OS in RCC patients; median OS: no irAEs 17.6 months; any irAEs not reached. Statistical analysis was performed using the log-rank test. HR, hazard ratio; CI, confidence interval; PFS: progression-free survival; OS, overall survival; irAE, immune-related adverse event.


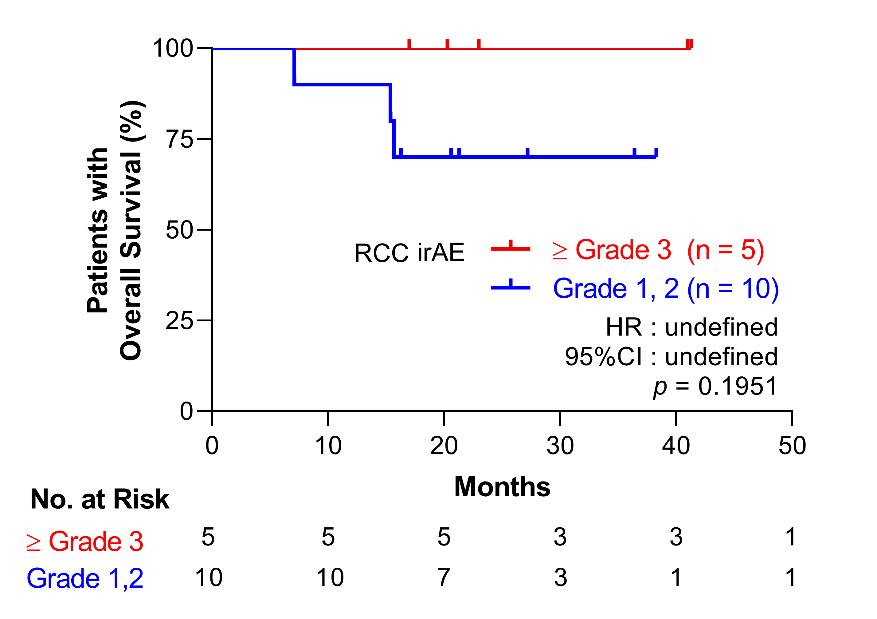

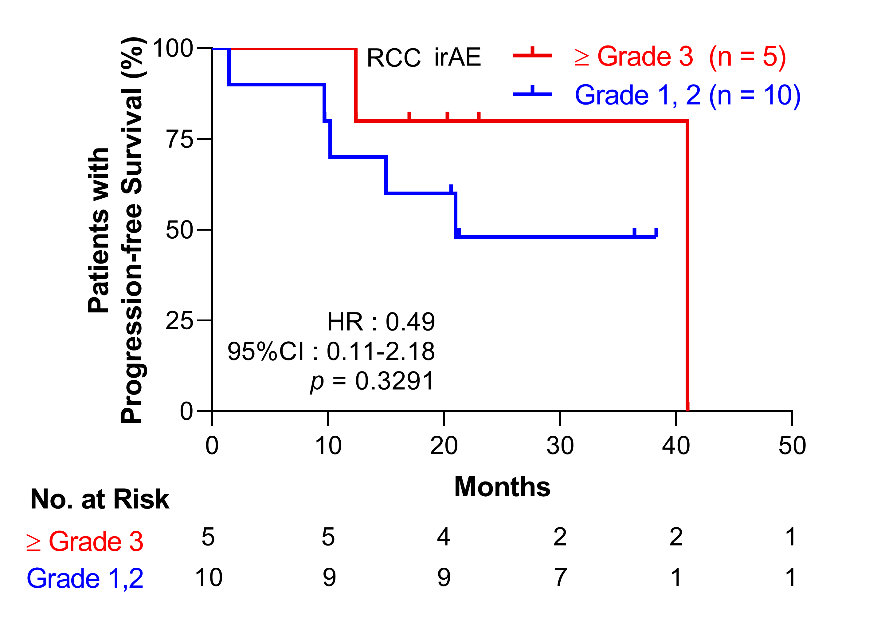

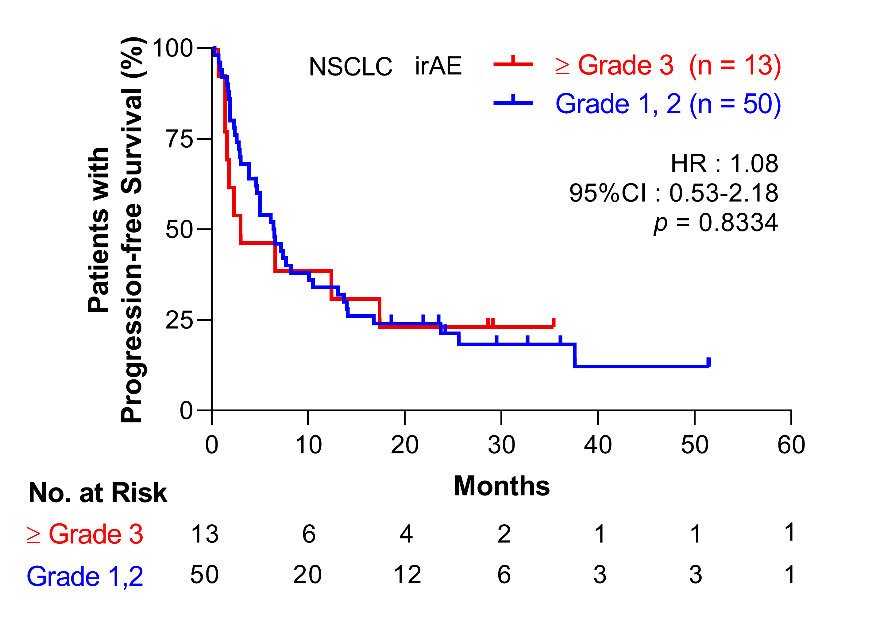

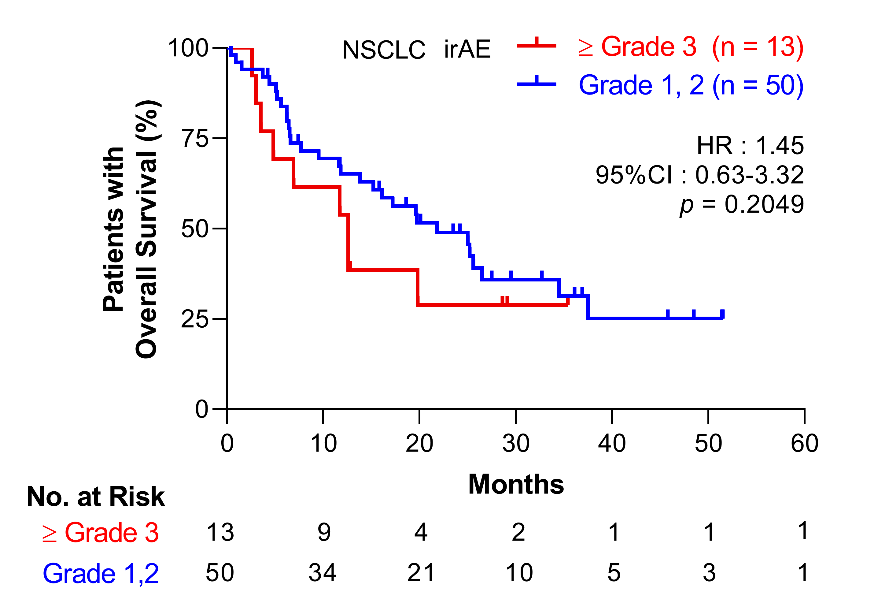

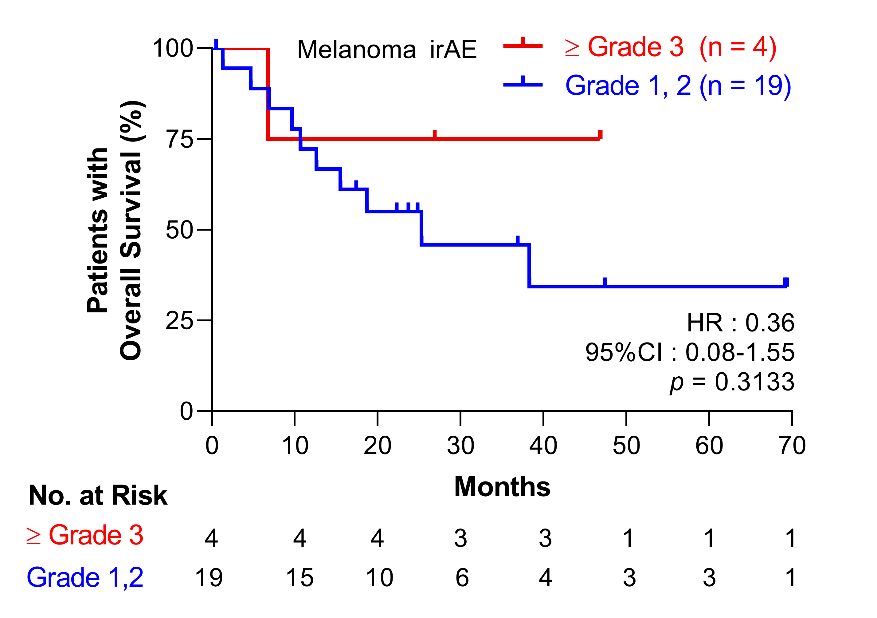

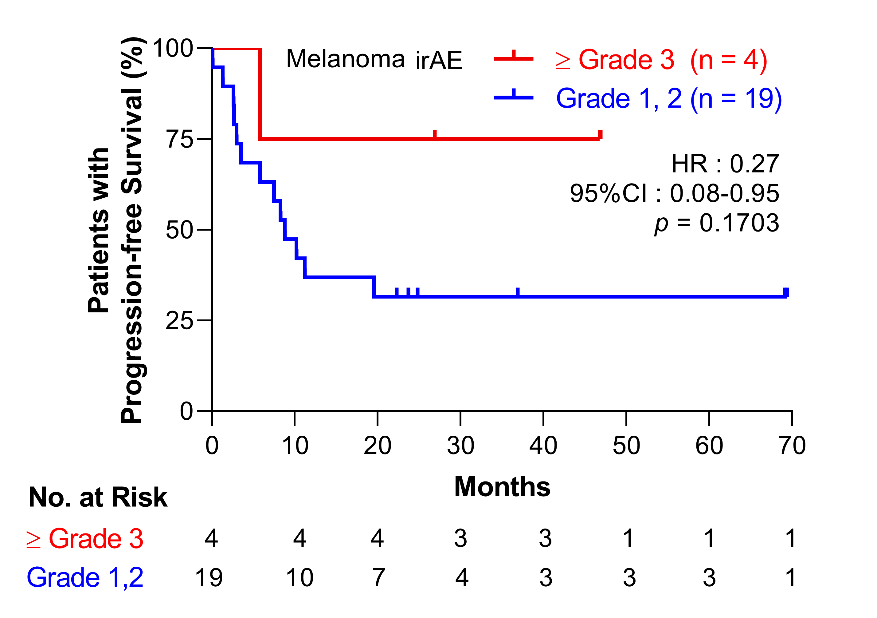

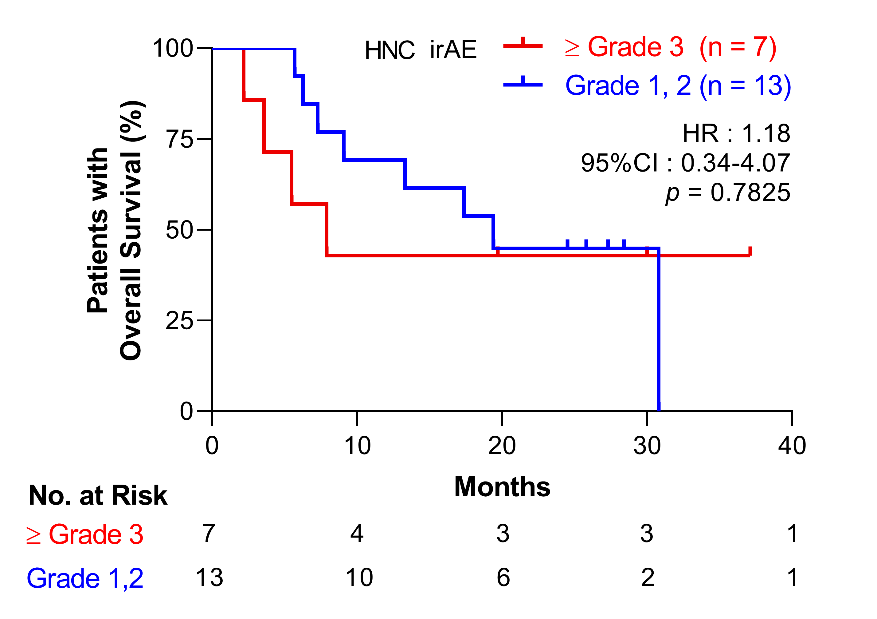

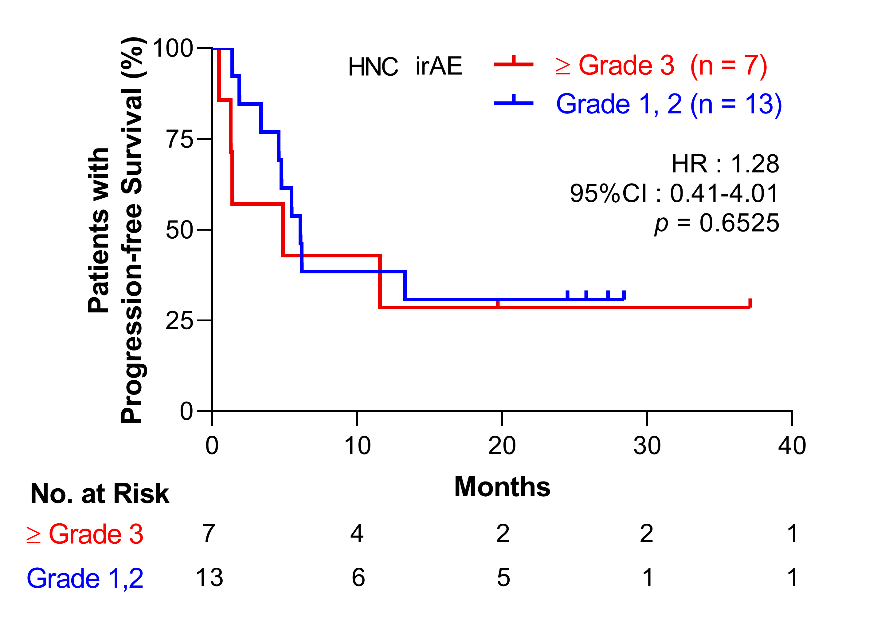


a

b

c

d

e

f

g

h

**Supplementary Fig. 5**.

**Kaplan-Meier survival analysis of PFS and OS according to the severity of an irAE.**

(**a**) PFS in melanoma patients; median PFS: Grade 1, 2 8.8 months, ≥Grade 3 not reached. (b) OS in melanoma patients; median OS: Grade 1, 2 25.3 months, ≥Grade 3 not reached. (c) PFS in non-small cell lung cancer (NSCLC) patients; median PFS: Grade 1, 2 6.5 months, ≥Grade 3 3.0 months. (d) OS in NSCLC patients; median OS: Grade 1, 2 21.8 months, ≥Grade 3 12.6 months. (e) PFS in head and neck cancer (HNC) patients; median PFS: Grade 1, 2 6.1 months, ≥Grade 3 4.9 months. (f) OS in HNC patients; median OS: Grade 1, 2 19.4 months, ≥Grade 3 7.9 months. (g) PFS in renal cell carcinoma (RCC) patients; median PFS: Grade 1, 2 21.0 months, ≥Grade 3 41.0 months. (h) OS in RCC patients; median OS: Grade 1, 2 not reached, ≥Grade 3 not reached. Statistical analysis was performed using the log-rank test. HR, hazard ratio; CI, confidence interval; PFS, progression-free survival; OS, overall survival; irAE, immune-related adverse event.
